# Artificial intelligence in respiratory medicine: From diagnosis to treatment and future directions

**DOI:** 10.1016/j.pccm.2026.05.005

**Published:** 2026-06-06

**Authors:** Zhe Chen, Zhiyong Tang, Rob M. Ewing, Zehor Belkhatir, Yihua Wang

**Affiliations:** a Biological Sciences, Faculty of Environmental and Life Sciences, University of Southampton, Southampton SO17 1BJ, UK; b Institute for Life Sciences, University of Southampton, Southampton SO17 1BJ, UK; c Electronics and Computer Science, Digital Health & Biomedical Engineering Group, University of Southampton, Southampton SO17 1BJ, UK; d NIHR Southampton Biomedical Research Centre, University Hospital Southampton, Southampton SO16 6YD, UK

**Keywords:** Lung diseases, Artificial intelligence, Deep learning, Imaging diagnostics, Multi-omics, Large language models, Digital twins, Precision medicine

## Abstract

Lung diseases—including lung cancer, chronic obstructive pulmonary disease (COPD), asthma, interstitial lung diseases (ILDs), and rare conditions like cystic fibrosis—remain major drivers of global morbidity and mortality. Timely diagnosis and individualized treatment are frequently challenged by heterogeneous clinical phenotypes and the complexity of multimodal data. This review provides a critical synthesis of the transformative role of artificial intelligence (AI) in respiratory care, tracing the paradigm shift from classical machine learning to emerging large language models (LLMs) and multimodal foundation models. We evaluate the performance of AI across the patient care continuum: beginning with radiologist-level nodule detection and automated diagnostics, advancing into AI-powered clinical decision support systems (CDSS) and surgical/radiotherapeutic interventions, and culminating in prognostic modeling and “digital twin” simulations for longitudinal patient management. Furthermore, we explore the translational frontier of precision medicine, examining how AI leverages multi-omics and liquid biopsies to drive novel biomarker discovery and accelerate drug repurposing. Finally, we address persistent sociotechnical barriers—including data sovereignty, legal liability, and the critical need for prospective clinical validation—proposing a translational roadmap for the safe integration of generalist medical AI into clinical workflows.

## Introduction

Lung diseases—encompassing chronic obstructive pulmonary disease (COPD), lung cancer, pneumonia, asthma, tuberculosis, and a spectrum of interstitial lung diseases (ILDs)—constitute a highly heterogeneous group of conditions that impose a profound and escalating global health burden.^1^ Pre-pandemic epidemiological assessments indicated that major respiratory conditions accounted for over eight million deaths annually,^2^ with this mortality disproportionately concentrated in low- and middle-income countries (LMICs).^3^ Furthermore, the systemic shocks of the coronavirus disease 2019 (COVID-19) pandemic starkly exposed the fragility of conventional, human-reliant respiratory care pathways, underscoring an urgent need for scalable, resilient, and highly accurate diagnostic solutions.

Within this critical context, artificial intelligence (AI) has emerged not merely as an instrument for computational automation, but as a paradigm-shifting force in pulmonary medicine.^4^ Historically, computational approaches in pulmonology relied heavily on classical machine learning (ML). While foundational, these early algorithms necessitated manual feature engineering—a labor-intensive process inherently constrained by human subjectivity and the inability to efficiently process high-dimensional unstructured data.

**Fig. 1:**
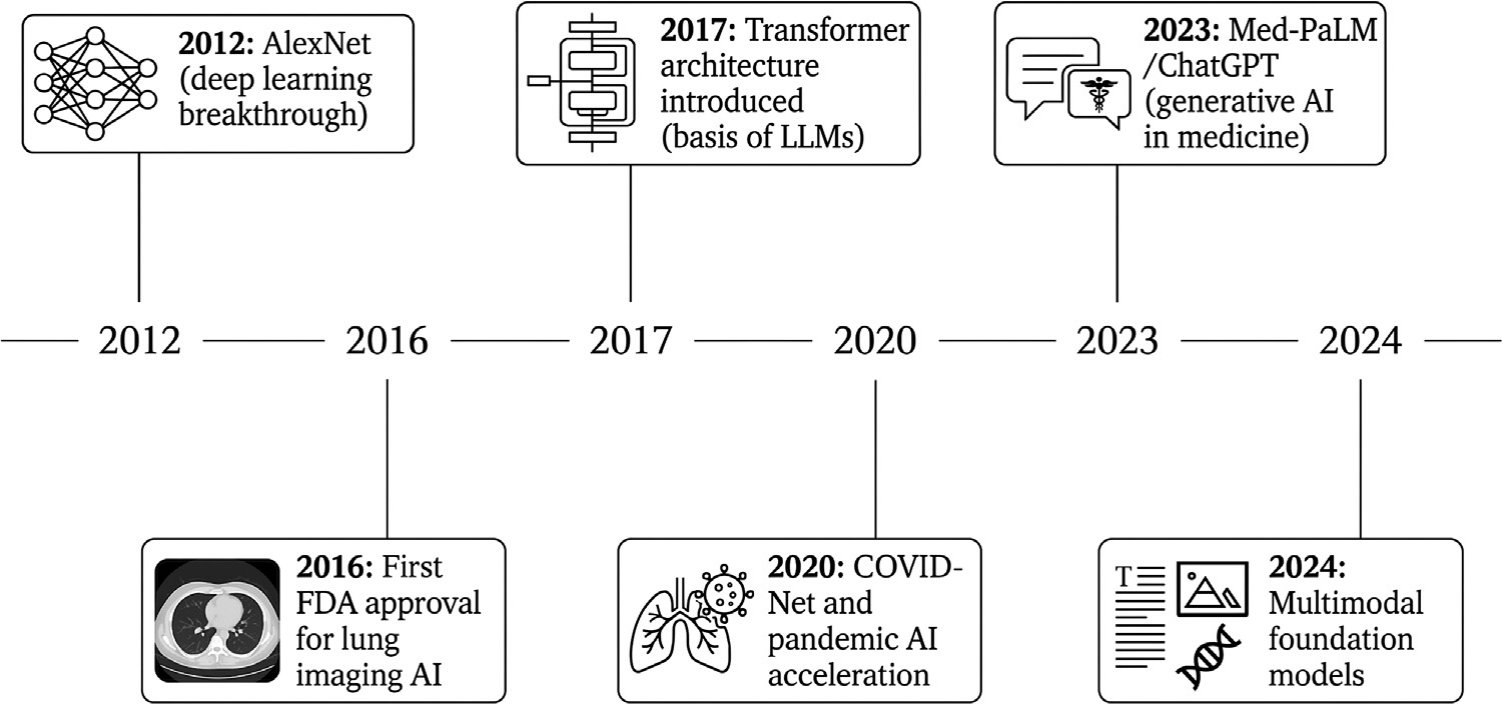
Timeline of key milestones in artificial intelligence relevant to pulmonary medicine, illustrating the rapid technological evolution from early deep learning breakthroughs in image recognition (AlexNet, 2012) to the first FDA approval for clinical lung imaging (2016). The timeline highlights the architectural paradigm shift brought by Transformers (2017), the accelerated deployment of AI during the pandemic (COVID-Net, 2020), and the recent emergence of medical-grade generative AI (Med-PaLM, 2023) and advanced multimodal foundation models (2024). AI, Artificial intelligence; FDA, Food and drug administration; LLMs, Large language models.

However, the past decade has witnessed an unprecedented technological evolution, moving the field from basic pattern recognition toward complex clinical reasoning.^5^, ^6^, ^7^ As illustrated in Fig. 1, this trajectory is marked by several key milestones. The breakthrough of AlexNet in 2012 catalyzed the deep learning (DL) era, proving that multilayered neural networks could autonomously extract salient features from radiographic inputs without manual intervention. This rapid maturation led to the first Food and Drug Administration (FDA) approvals for lung imaging AI in 2016. Subsequently, the introduction of the transformer architecture in 2017 laid the indispensable groundwork for modern natural language processing (NLP). The 2020 pandemic acted as a global accelerant, resulting in the rapid deployment of diagnostic networks like COVID-Net. Most recently, the landscape has been redefined by the 2023 introduction of medically tuned large language models (LLMs) such as Med-PaLM, culminating in the 2024 shift toward true multimodal foundation models capable of synthesizing diverse data streams.

This technological maturation has facilitated a critical convergence of data modalities. While convolutional neural networks (CNNs) have mastered pixel-based tasks—frequently matching or surpassing human radiologist performance in the detection of pneumonia, emphysema, and pulmonary nodules,^8^^,^^9^ NLP technologies have proven equally instrumental in parsing free-text clinical documents. By transforming unstructured narrative from radiology and pathology reports into structured, quantifiable data (as visualized in Supplementary Fig. 1), NLP effectively bridges the gap between raw pixel data and broader clinical contexts.^10^^,^^11^

Simultaneously, AI algorithms are expanding beyond imaging and text. The deployment of AI for respiratory sound analysis and spirometry interpretation is enabling the remote monitoring and early detection of chronic airway diseases, fostering a shift toward proactive self-management.^12^ Furthermore, the AI-enabled analysis of multi-omics data (genomics, transcriptomics, and proteomics) is rapidly advancing biomarker discovery and molecular subtyping for complex disorders like idiopathic pulmonary fibrosis and severe asthma.

The clinical implementation of these technologies, however, reveals a stark geographic dichotomy. In high-income regions such as the United States and Europe, regulatory bodies (*e.g.,* the FDA and European Medicines Agency [EMA]) have cleared over 500 AI algorithms. Here, platforms like Viz.ai and Riverain have been seamlessly integrated into lung cancer screening workflows to optimize efficiency and diagnostic yield. Conversely, in LMICs, AI adoption is primarily driven by acute resource scarcity. In regions across India and Sub-Saharan Africa, automated tools like qXR are widely deployed to screen chest X-rays for tuberculosis, effectively compensating for critical shortages of specialized radiologists.^8^^,^^9^

Ultimately, these advancements herald a profound paradigm shift: AI is increasingly embedded within clinical decision support systems (CDSS) that equip pulmonologists to optimize treatment strategies, stratify patient risk, and allocate scarce resources with unprecedented efficacy.^13^ By simulating complex clinical reasoning, these systems deliver personalized, evidence-based recommendations, propelling respiratory care toward true precision medicine.^14^^,^^15^ A comprehensive overview of these foundational AI architectures and their specific clinical relevance is provided in Table 1.

**Table 1:** Key artificial intelligence (AI) concepts and architectures relevant to lung disease diagnosis.

Evolutionary category	Architecture/Model	Description & clinical relevance in pulmonary care	Reference (s)
Classical machine learning (ML)	Logistic regression (LR)	A baseline statistical model for binary classification, commonly used for tuberculosis detection and mortality risk stratification.	^16^
	Support vector machines (SVM)	High-dimensional classifiers applied to gene expression data, spirometry-based screening, and respiratory acoustic signals.	^17^
	k-nearest neighbors (k-NN)	A non-parametric method used for symptom clustering and acoustic similarity modelling in cough analysis.	^18^
	Random forests (RF)	An ensemble learning method providing robust clinical risk prediction and radiomics-based detection of lung cancer.	^19^
Deep learning (DL)	Convolutional neural networks (CNN)	The foundational architecture for medical imaging; used for lung segmentation, nodule detection, and pneumonia classification.	^9^
	Recurrent neural networks (RNNs)/long short-term memory (LSTM) /gated recurrent unit (GRU)	Specialized for sequential data; applied to longitudinal pulmonary function test (PFT) trends and real-time waveform monitoring.	^20^
	U-Net	A specialized CNN architecture designed for precise medical image segmentation, such as delineating fibrosis boundaries or airway structures.	^21^
	Residual networks (ResNet)	Deep CNN variants that improve feature extraction for complex patterns in digital pathology and interstitial lung disease (ILD).	^22^
	Transformers (*e.g.,* vision transformer (ViT), swin transformer (SwinT)	Attention-based models capable of handling global context in high-resolution computed tomography (CT) scans for coronavirus disease 2019 (COVID-19) and ILD classification.	^23^
Generative AI & large language models (LLMs)	Generative adversarial networks (GANs)	Generative models used for synthetic data augmentation to train algorithms on rare pulmonary pathologies.	^24^
	Bidirectional encoder representations from transformers for clinical notes or biomedical text mining (ClinicalBERT/BioBERT)	Domain-specific language models used to extract structured information from unstructured radiology and pathology reports.	^25^
	Large language models (LLMs) (*e.g.,* generative pre-trained transformer-4 [GPT-4])	Advanced generative models used for report summarization, diagnostic reasoning, and multi-modal clinical decision support.	^26^
	Multimodal foundation models (*e.g.,* contrastive language–image pre-training [CLIP])	The current frontier of AI; these models align diverse data types, such as CT images, pathology slides, and clinical text, for holistic analysis.	^27^

To fully appreciate the translational impact of AI across the respiratory care continuum, it is essential to understand the distinct algorithmic architectures that underpin these clinical applications. As detailed in Table 1, the technological evolution of medical AI can be broadly categorized into three progressive epochs: classical machine learning (ML), DL, and generative artificial intelligence.

### Classical ML: The era of feature engineering

Early computational approaches in pulmonary medicine were driven by classical ML algorithms, which rely heavily on structured clinical data and manually extracted features (*e.g.,* radiomics). Baseline statistical models like logistic regression (LR) remain fundamental for binary classification tasks, such as automated tuberculosis detection and baseline mortality risk stratification.^16^ For more complex, high-dimensional datasets—such as gene expression arrays or respiratory acoustic signals—support vector machines (SVM) are frequently deployed.^17^ Furthermore, non-parametric methods like k-nearest neighbors (k-NN) have shown utility in symptom clustering and acoustic similarity modeling for cough analysis,^18^ while ensemble learning methods like random forests (RF) provide robust performance in clinical risk prediction and early radiomics-based detection of lung cancer.^19^ Despite their interpretability, these classical models are inherently limited by their dependence on laborious manual feature extraction.

### DL: Mastering unstructured clinical data

The limitations of classical ML were largely overcome by DL, a subset of AI utilizing multilayered neural networks to autonomously extract salient features from raw, unstructured data. CNN represent the foundational architecture for medical imaging, achieving expert-level accuracy in lung nodule detection and pneumonia classification.^9^ Specialized CNN variants have further refined these capabilities: U-Net architectures are now the gold standard for precise medical image segmentation, adeptly delineating fine structures such as fibrosis boundaries or airway walls,^20^ while deep ResNet excels in extracting complex pathological patterns from digital whole-slide images.^22^ Beyond spatial imaging, recurrent architectures like recurrent neural networks (RNN), long short-term memory (LSTM), and gated recurrent units (GRU) are specifically designed for sequential data. These models are critical for monitoring time-series physiological signals, such as longitudinal pulmonary function test (PFT) trends and real-time ventilator waveforms.^22^ More recently, vision-based transformers (*e.g.,* vision transformer [ViT], swin transformer [SwinT] have been introduced to handle global contextual dependencies in high-resolution computed tomography (CT) (HRCT) scans, offering superior classification performance for spatially diffuse conditions like COVID-19 and ILDs.^23^

### Generative AI, LLMs, and foundation models: The current frontier

The most recent and disruptive paradigm shift is the advent of generative AI and LLMs. A critical challenge in pulmonary AI—particularly for rare diseases like cystic fibrosis or pulmonary alveolar proteinosis—is data scarcity. Generative adversarial networks (GANs) address this by creating high-fidelity synthetic data to augment training cohorts.^24^ In the realm of clinical text, domain-specific language models like bidirectional encoder representations from transformers for clinical notes or biomedical text mining (ClinicalBERT/BioBERT) have revolutionized the extraction of structured phenotypes from unstructured radiology and pathology reports.^25^ Building upon this, advanced LLMs (*e.g.,* generative pre-trained transformer 4 [GPT-4]) are now capable of sophisticated diagnostic reasoning, report summarization, and interactive clinical decision support.^26^ Ultimately, the field is converging toward multimodal foundation models (*e.g.,* contrastive language–image pre-training [CLIP]). By mapping diverse data types—such as CT pixels, pathology slides, and clinical narrative text—into a unified representational space, these models enable the holistic, cross-modal analysis required for true precision respiratory medicine.^27^

This review presents a comprehensive synthesis of the current landscape and future trajectories of artificial intelligence across pulmonary medicine. To accurately reflect the translational impact of these technologies, this paper structurally mirrors the clinical patient journey from macro-level clinical management to micro-level molecular biology. We first evaluate the transformative role of AI in diagnostic imaging, followed by its integration into treatment planning and decision support. We then examine AI’s impact on longitudinal patient management and prognostic modeling. Moving from the clinical environment to underlying molecular mechanisms, we subsequently explore the frontier of multi-omics, liquid biopsies, and AI-driven drug discovery. Finally, we systematically address the persistent sociotechnical barriers and outline future directions, including FL and foundation models.

## AI in diagnostic imaging

**Fig. 2:**
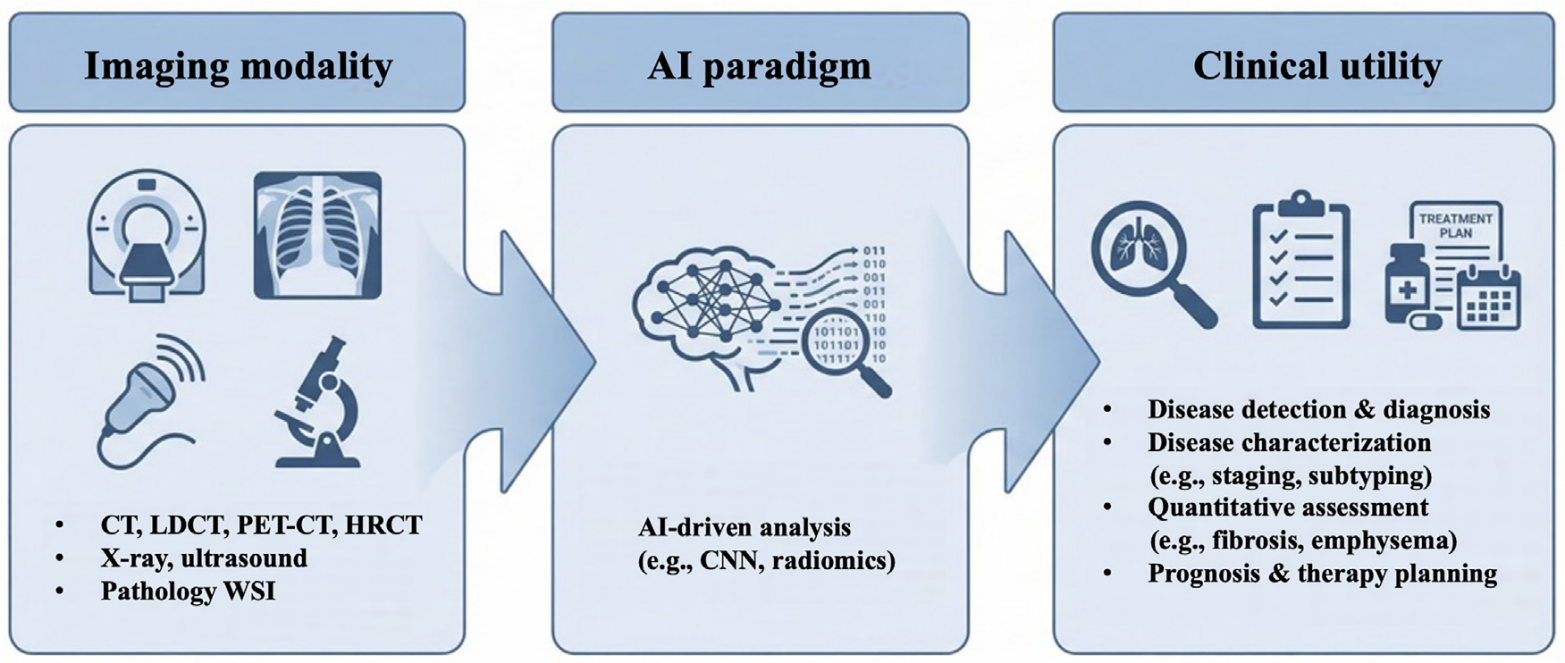
A simplified framework for AI-driven diagnostics in respiratory medicine. This high-level flow diagram illustrates the transformation of various medical imaging modalities (*e.g.,* CT, X-ray, ultrasound, pathology WSI) into clinical utility through AI-driven analysis. The “AI Paradigm” stage, employing techniques like CNN and radiomics, processes the input data. This leads to key clinical applications, including disease detection and diagnosis, disease characterization (*e.g.,* staging, subtyping), quantitative assessment (*e.g.,* fibrosis, emphysema), and support for prognosis and therapy planning. This simplified view is presented to reduce clutter and enhance clarity. AI, Artificial intelligence; CNN, Convolutional neural network; CT, Computed tomography; HRCT, High-resolution computed tomography; LDCT, Low-dose computed tomography; PET-CT, Positron emission tomography-computed tomography; WSI, Whole slide imaging.

The integration of AI into clinical decision-making has conclusively transitioned from experimental automation to a paradigm-shifting force in pulmonary medicine. Rather than viewing diagnostic tools in isolation, contemporary research emphasizes holistic diagnostics—moving beyond isolated, task-specific models toward comprehensive systems that bridge raw pixel data with the biological and pathophysiological foundations of respiratory disease.^28^ High-resolution imaging and next-generation sequencing platforms now routinely generate voluminous, multidimensional data, and AI systems are uniquely equipped to interrogate this complexity to facilitate the discovery of novel sub-visual biomarkers and early disease indicators long before clinical manifestation. Underpinned by advanced computational modeling and bioinformatics, AI has fundamentally transformed the diagnostic landscape by enabling highly sensitive interpretations across a broad spectrum of modalities, ranging from HRCT to digital whole-slide imaging (WSI) in histopathology. The integration of these diverse modalities with robust deep learning architectures has inaugurated an era of multimodal, data-rich diagnostic workflows that transcend the inherent limitations of human visual perception. As illustrated in Fig. 2, this modern diagnostic framework operates through a tripartite pipeline: diverse imaging modalities capture multiscale anatomical and cellular data; an AI paradigm processes these inputs using convolutional neural networks (CNNs) and radiomics; and the computational engine translates the data into direct clinical utility spanning disease detection, quantitative assessment, and prognostic planning.^29^ A comprehensive summary of the specific AI diagnostic tools currently redefining these clinical benchmarks is detailed in Table 2.

**Table 2:** Imaging-based AI diagnostics in lung diseases.

AI diagnostic tool	Lung disease focus	Imaging modality	Core AI model(s) used	Dataset	Benchmark	Clinical validation status	Limitations/challenges	Reference(s)
DeepLung	Lung cancer, pulmonary nodules	LDCT	3D CNN, radiomics	LIDC-IDRI, LUNA16	Sensitivity: 90.1 %; specificity: 90.4 %	Validated (retrospective, public datasets)	Nodule variability, generalizability	^45^^,^^46^
CHIEF	Lung cancer, subtyping	Digital pathology (WSI)	Transformer-based DL	>60,000 WSIs	AUROC: 0.94 (subtyping); AUROC: 0.96 (tumor detection)	Multi-center validation	High computational cost	^36^
Prov-GigaPath	Lung cancer, subtyping	Digital pathology (WSI)	Self-supervised + Transformer	>30,000 WSIs (US/EU/Asia)	AUROC: 0.93–0.97 across various subtyping tasks (SOTA)	Research use; external validation	Data bias, annotation quality	^47^^,^^48^
PET-LungAI	Lung cancer staging	PET-CT	Radiomics + deep learning	NLST, institutional datasets	Accuracy: 88.5 %; AUROC: 0.91; sensitivity: 86.2 %	Clinical research/pilot use	High imaging cost, limited accessibility	^32^^,^^33^
AI-BronchoVision	Early airway tumors	Bronchoscopy video	YOLO + ResNet	Annotated bronchoscopy videos	Sensitivity: 92.5 %; PPV: 85.3 %; accuracy: 89.2 %	Pilot studies	False positives, lighting artifacts	^30^^,^^31^
Medpseg/ResNet for FBA	Radiolucent foreign body aspiration	HRCT	Medpseg + ResNet	Institutional dataset	Sensitivity: 91.0 %; specificity: 94.0 %; accuracy: 92.5 %	Multi-center validation	Small dataset	^40^
YOLO/ResNet for Bronchoscopy	Airway tumors, mucosal lesions	Bronchoscopy video	Real-time object detection CNN	Institutional datasets, live procedures	F1-score: 0.88; mAP: 0.85; sensitivity: 90.0 %	Clinical pilot; decision support	Video quality, operator trust	^31^
qXR	Tuberculosis, pneumonia	Chest X-ray	2D CNN	ChestXray14, India/Africa clinics	Sensitivity: 95.0 %; specificity: 90.0 %; NPV: 99.0 %	Widely deployed (India, Africa clinics)	Overfitting in small TB datasets	^9^^,^^49^
COVID-Net, RSNA COVID Challenge	COVID-19, viral pneumonia	X-ray, CT	Ensemble CNN, explainable AI	RSNA COVID Challenge, open datasets	Accuracy: 93.3 %; sensitivity: 91.0 %; PPV: 95.2 %	Global deployment during pandemic	Variability in data, rapid deployment	^50^^,^^51^
TD-CNN-LSTM-LungNet	Pneumonia, pulmonary edema	Lung ultrasound (video)	Explainable 2D CNN + LSTM	Custom lung US datasets	Accuracy: 96.57 %; F1-score: 0.96; AUROC: 0.98	Research phase (not yet commercial)	Generalizability, video quality	^43^^,^^52^
COPDGene AI Analysis	COPD, emphysema subtyping	Quantitative CT	CNN + ML, radiomics	COPDGene study, ECLIPSE, SPIROMICS	Accuracy: 86.0 % for phenotype classification; AUROC: 0.88	Used in cohort research, influencing guidelines	Data harmonization, phenotype variability	^38^^,^^39^
VUNO Med–LungCT AI	ILDs, lung cancer	HRCT	CNN + radiomics	Multi-center Asian data	AUROC: 0.92–0.95 (ILD classification); accuracy: 90.0 %	Approved for clinical use (Korea)	Expert interpretation required	^33^^,^^53^
AI-CTPA	Pulmonary embolism	CT pulmonary angiography	Deep CNN (2D/3D)	Institutional, open CTPA datasets	Sensitivity: 92.6 %; specificity: 95.8 %; NPV: 98.1 %	Increasing clinical deployment, ED use	False positives in low-prevalence settings	^41^^,^^42^
EndoLung-Net	Dysplasia, endobronchial lesions	Endoscopy (NBI, WLI)	DenseNet, explainable DL	Clinical images (Europe/Asia)	Accuracy: 92.4 %; sensitivity: 91.5 %; specificity: 93.2 %	Research phase, multi-site	Device dependency, generalizability	^54^
MRI-PulmoDL	Pediatric lung tumors, soft tissue	MRI	CNN	Institutional pediatric MRI datasets	Dice coefficient: 0.89; accuracy: 91.0 %; AUROC: 0.93	Early validation, academic centers	Low MRI adoption for thoracic disease	^34^^,^^35^

AI, Artificial intelligence; AUROC, Area under the receiver operating characteristic curve; CHIEF, Clinical histopathology imaging evaluation foundation; CNN, Convolutional neural network; COPD, Chronic obstructive pulmonary disease; COVID-19, Coronavirus disease 2019; CT, Computed tomography; CTPA, Computed tomography pulmonary angiography; DL, Deep learning; ECLIPSE, Evaluation of COPD longitudinally to identify predictive surrogate endpoints; ED, Emergency department; EU, European Union; FBA, Foreign body aspiration; HRCT, High-resolution computed tomography; ILDs, Interstitial lung diseases; LDCT, Low-dose computed tomography; LIDC-IDRI, Lung image database consortium and image database resource initiative; LSTM, Long short-term memory; LUNA16, Lung nodule analysis 2016; mAP, Mean average precision; ML, Machine learning; MRI, Magnetic resonance imaging; NPV, Negative predictive value; NBI, Narrow-band imaging; NLST, National lung screening trial; PET, Positron emission tomography; PPV: Positive predictive value; ResNet, Residual network; RSNA, Radiological society of North America; SOTA, State-of-the-art; SPIROMICS, Subpopulations and intermediate outcome measures in COPD study; TB, Tuberculosis; US, Ultrasound/United States; WLI, White-light imaging; WSI, Whole slide image.

### Lung cancer detection and characterization

The application of AI in thoracic oncology represents the most mature integration of computational technologies in clinical practice, shifting strategically from mere detection to comprehensive characterization. In macroscopic imaging, DL systems have established a new clinical standard for early screening. Platforms such as DeepLung (Table 2) leverage three-dimensional convolutional neural networks (3D-CNNs) alongside radiomics on low-dose computed tomography (LDCT) scans. Validated retrospectively on prominent public datasets including lung image database consortium and image database resource initiative (LIDC-IDRI) and lung nodule analysis 2016 (LUNA16), DeepLung consistently achieves sensitivities exceeding 90 %, although it faces persistent clinical challenges regarding nodule variability and cross-cohort generalizability.^30^^,^^31^ Beyond simple localization, AI-enabled radiomics quantifies sub-visual topography—such as textural heterogeneity, sphericity, and perinodular vascularity—allowing clinicians to accurately differentiate between indolent lesions and aggressive malignant nodules to reduce unnecessary invasive biopsies. To further refine tumor staging, hybrid models like PET-LungAI integrate DL with positron emission tomography-computed tomography (PET-CT) radiomics.^32^^,^^33^ Trained on data from the national lung screening trial (NLST) alongside institutional datasets, this model captures sub-visual metabolic heterogeneity to improve diagnostic accuracy versus standard radiologist assessments; however, high imaging costs and limited accessibility restrict it primarily to clinical research and pilot use. Furthermore, models like MRI-PulmoDL demonstrate the emerging utility of CNNs in delineating pediatric lung tumors and soft tissue margins.^34^^,^^35^ Evaluated using institutional pediatric MRI datasets, this tool offers improved margin detection and circumvents ionizing radiation risks, though it currently remains in early validation at academic centers due to historically low MRI adoption for thoracic diseases. At the cellular microscopic level, the advent of digital pathology has catalyzed profound breakthroughs in lung cancer subtyping. Leveraging Transformer-based DL architectures, foundational models like clinical histopathology imaging evaluation foundation (CHIEF) (Table 2) have been trained on over 60,000 whole-slide images (WSIs) to achieve an exceptional area under the receiver operating characteristic curve (AUROC) of greater than 0.93.^36^ While currently undergoing multi-center validation, its universal deployment is hindered by substantial computational costs. Similarly, the Prov-GigaPath model utilizes a self-supervised Transformer framework trained on an extensive dataset of over 30,000 WSIs from the United States, Europe, and Asia. Delivering state-of-the-art AUROC for tumor subtyping, it is currently restricted to research use while developers address critical translational limitations surrounding dataset bias and annotation quality.

To bridge the gap between screening and intervention, AI is now integrated directly into live diagnostic procedures. In interventional settings, platforms like AI-BronchoVision and specialized YOLO/ResNet architectures deploy real-time object detection on live bronchoscopy video feeds. These tools highlight suspicious mucosal lesions and early airway tumors during the procedure, enhancing the precision of biopsy targeting. Similarly, EndoLung-Net leverages DenseNet and explainable deep learning to identify dysplasia and endobronchial lesions. Specifically, it has demonstrated an accuracy greater than 92 % in the early-stage identification and classification of neoplastic lesions using narrow-band and white-light endoscopic images.^37^ While promising, its ultimate clinical translation depends on overcoming device dependency and improving imaging generalizability across distinct patient populations.

### ILD and COPD phenotyping

In the context of chronic and fibrotic conditions, AI is shifting the clinical focus toward intricate, quantitative subtyping. For interstitial lung diseases (ILDs), AI-powered quantitative CT analysis has become an indispensable adjunct to multidisciplinary team (MDT) assessments. DL models autonomously identify structural hallmarks such as reticulation, honeycombing, and ground-glass opacities, which facilitates highly accurate differential diagnoses among idiopathic pulmonary fibrosis (IPF), nonspecific interstitial pneumonia (NSIP), and hypersensitivity pneumonitis. For instance, the VUNO Med–LungCT AI platform combines CNNs with radiomic features to analyze HRCT scans. Validated on multi-center Asian data, this model provides improved classification between ILDs and lung cancer and is approved for clinical use in Korea, though it still necessitates downstream expert interpretation. Similarly, in COPD, AI-enhanced quantitative CT enables the detailed phenotyping of small airway narrowing and regional air trapping. The COPDGene AI Analysis framework utilizes CNNs, machine learning, and radiomics to discover novel emphysema phenotypes based on extensive cohorts like the COPDGene study, ECLIPSE, and SPIROMICS.^38^^,^^39^ Actively used in cohort research and directly influencing clinical guidelines, this framework successfully stratifies patients into specific exacerbation-risk phenotypes, although complex challenges related to multi-center data harmonization and physiological phenotype variability remain.

### Acute, infectious, and dynamic diagnostics

Beyond chronic disease management, AI is increasingly deployed to resolve diagnostically complex, time-sensitive acute conditions and infectious diseases, acting as a critical equalizer for global health. In the realm of public health, tools like qXR (Table 2) employ robust 2D-CNNs to interpret chest X-rays for tuberculosis and pneumonia screening. Trained on the ChestXray14 database and clinical data from clinics in India and Africa, qXR achieves 95 % sensitivity and 90 % specificity.^9^^,^^40^ It has been widely deployed in resource-limited clinics to offset critical radiologist shortages, though developers note the risk of model overfitting when utilized on smaller, localized tuberculosis datasets. Similarly, the global deployment of COVID-Net during the pandemic highlighted the capacity of ensemble CNNs and explainable AI for the high-throughput, robust severity grading of viral pneumonia using both X-ray and CT modalities. Utilizing data from the RSNA COVID Challenge and open datasets, this tool facilitated rapid triage, despite encountering generalizability challenges caused by rapid deployment and global data variability. Furthermore, AI excels in identifying cryptic acute conditions. Radiolucent foreign body aspiration (FBA), for example, is notoriously subtle and historically led to high misdiagnosis rates on standard chest CTs. A recent DL model combining high-precision airway segmentation (Medpseg) with a ResNet classifier on institutional HRCT datasets significantly outperformed expert radiologists, achieving a recall of 71.4 % versus 35.7 % and an F1 score of 74.1 % versus 52.6 %.^41^ While demonstrating robust clinical promise, this tool requires rigorous multi-center validation to overcome the limitations of a small initial training dataset. Concurrently, AI-assisted CT pulmonary angiography (AI-CTPA) platforms utilizing deep 2D and 3D CNNs are rapidly analyzing CT pulmonary angiography to accurately detect emboli and assess right ventricular strain.^40^^,^^42^ Validated on both institutional and open datasets, these platforms are seeing increasing clinical deployment in emergency departments to accelerate triage, although mitigating false positives in low-prevalence settings remains a clinical hurdle. Finally, AI is advancing the real-time analysis of spatiotemporal dynamic imaging by processing temporal sequences rather than static frames. Hybrid models such as TD-CNN-LSTM-LungNet apply explainable 2D-CNNs combined with long short-term memory (LSTM) networks to point-of-care lung ultrasound (POCUS) videos. This architecture enables the model to capture the dynamic “sliding sign” of the pleura and rhythmic lung artifacts over time, successfully differentiating between cardiogenic pulmonary edema and viral pneumonia with 96.57 % accuracy.^43^ Such capability allows for continuous, bedside monitoring of pulmonary aeration changes, which is crucial for guided recruitment maneuvers and rapid triage in intensive care environments.^44^

## AI in treatment planning and decision support

**Fig. 3:**
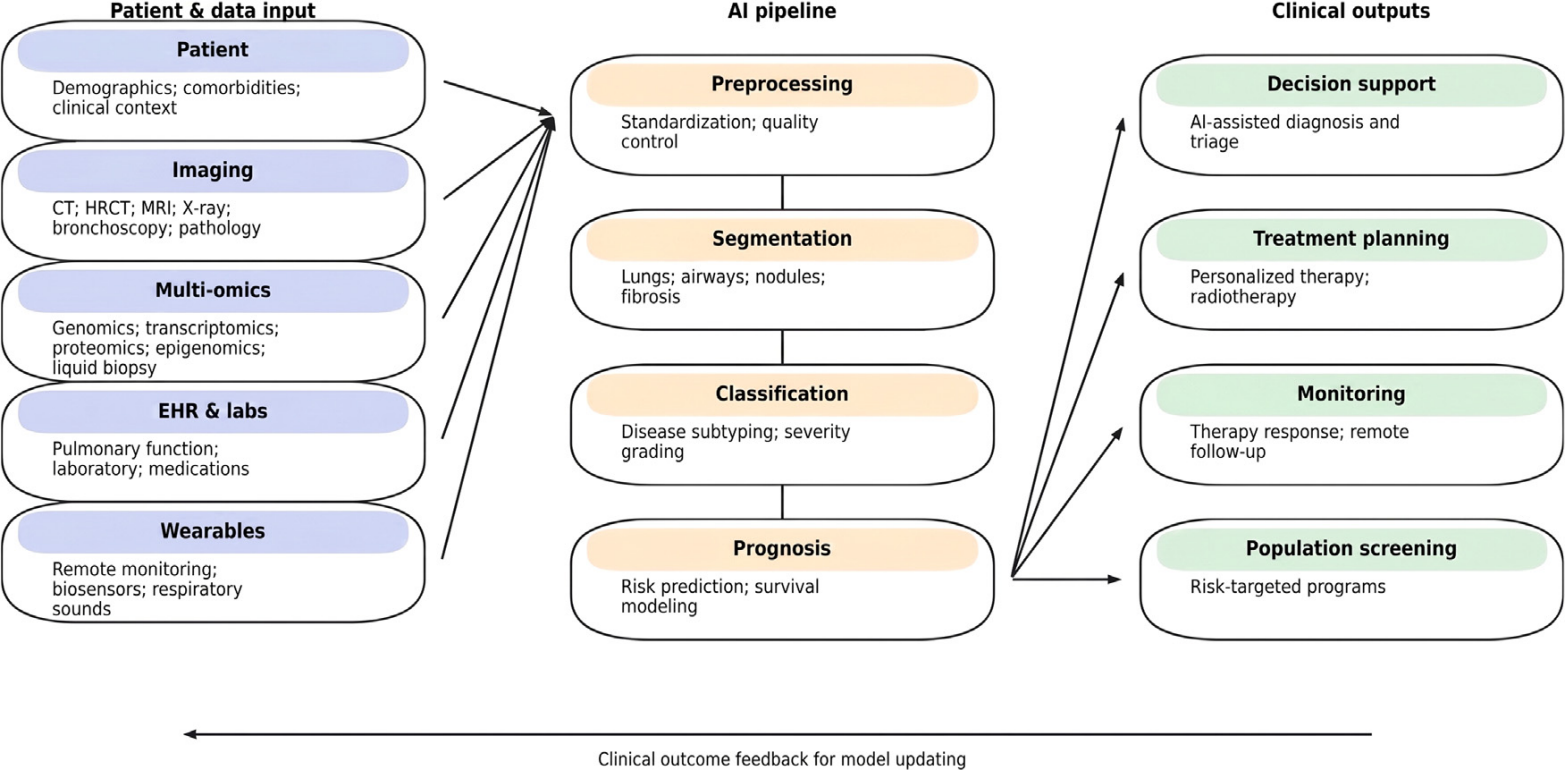
Evolution of AI-powered clinical decision support workflow for lung disease management. The framework illustrates the hierarchical transformation of multimodal inputs—including imaging, multi-omics, EHRs, and wearable data—into clinical intelligence. While the central AI pipeline depicts foundational deep learning tasks (such as segmentation and classification), these components now serve as the structural backbone for emerging multimodal foundation models and medical LLMs. By integrating these modular outputs, modern systems facilitate cross-modal reasoning, allowing for generative decision support, explainable treatment rationales, and real-time monitoring. This workflow reflects the broader paradigm shift from narrow task-specific algorithms toward generalist medical AI (GMAI), where raw biomedical data are synthesized into interactive and actionable insights for precision respiratory medicine. AI, Artificial intelligence; CT, Computed tomography; EHR, Electronic health record; HRCT, High-resolution computed tomography; LLMs, Large language models; MRI, Magnetic resonance imaging***.***

Over the past decade, the landscape of treatment planning and clinical decision support in pulmonary medicine has been fundamentally transformed. AI-driven platforms, underpinned by advances in deep learning, probabilistic modeling, and large-scale data integration, now empower clinicians to synthesize multidimensional data—including high-resolution imaging, genomics, and real-world longitudinal clinical records. This therapeutic paradigm is currently undergoing a shift from discriminative “black-box” classifiers toward generative AI and multimodal foundation models.^55^ AI-powered CDSS are particularly impactful in pulmonology, where management strategies must constantly adapt to complex interactions between heterogeneous disease phenotypes, comorbidities, and rapidly evolving therapeutic guidelines.^56^ As conceptualized in the updated operational workflow (Fig. 3), the traditional DL pipeline—encompassing data preprocessing, segmentation, and classification—now functions as a modular component within broader, LLM-driven reasoning frameworks. These advanced systems utilize medical LLMs to perform complex, high-level tasks such as automated report summarization, cross-modal diagnostic reasoning, and the synthesis of individualized therapeutic strategies based on real-time evidence. By simulating expert-level clinical reasoning, these models act as interactive “clinical co-pilots,” providing explainable recommendations that bridge the gap between raw data acquisition and actionable, patient-specific management. To fully appreciate the clinical integration of these systems, Table 3, Table 4 delineate representative AI platforms currently deployed across various respiratory and interventional domains, highlighting their specific data inputs, core architectures, and clinical benchmarks.

**Table 3:** Representative AI-powered decision support systems in lung disease management.

AI system/ platform	Disease focus	Input data sources	Core AI approach	Clinical application areas	Clinical impact/ benchmark	Challenges/ limitations	Reference(s)
Tempus, OncoKB, Watson	Lung cancer, all-stage	Genomics, pathology, imaging, EHR	Ensemble ML, NLP	Targeted/ Immunotherapy selection, clinical trial matching, molecular tumor boards decision support	Up to 80 % concordance with expert tumor board consensus; AUC 0.85–0.92 for treatment response	Cost, high computational demand, model transparency	^58^
Qure.ai, Arterys, RayStation, Varian Ethos	Lung cancer, RT planning	CT, MRI, PET-CT tomography, radiomics	Deep learning (CNN, U-Net, transformers)	Automated segmentation; dose adaptation; toxicity prediction	>90 % DSC for OARs; 30 % reduction in planning time	Device dependency, QA integration	^36^^,^^59^
COPDGene-AI, SPIROMICS-CDSS	COPD	EHR, spirometry, imaging, biomarker	Random forest, LSTM	Exacerbation prediction, inhaler optimization, noninvasive ventilation use	AUROC 0.85 for 30-day exacerbation risk; 15–30 % reduction in hospitalizations	Data harmonization, generalizability	^60^^,^^61^
AsthmaPredict, GENEVA-AI	Asthma (adult, paediatric)	Spirometry, sensors, EHR, environment	Multimodal DL, XAI	Biologic therapy selection; adherence monitoring; exacerbation risk	AUC 0.89–0.93 for phenotype classification; improved control in RCTs	Lack of large-scale multicenter pediatric cohorts; sensor noise	^62^^,^^63^
ILD-RiskNet, LUNGOMIC-AI	ILD (e.g. IPF, NIP)	HRCT, PFT, omics, adherence data	SVM, CNN, multimodal fusion	Antifibrotic selection, transplant referral, progression tracking	AUROC >0.85 for 1-year progression prediction; sensitivity >88 % for rapid decliners	Multimodal data alignment, cost, rarity of specific ILD subtypes	^64^^,^^65^
ARDS-CDSS, COVID-TriageAI	ARDS, pneumonia, COVID-19	EHR, Bedside monitoring, physiological signals	DL, real-time analytics	Triage, ventilator management, infection control	20 % improvement in AUROC for mortality prediction *vs*. traditional SOFA scores	Real-time data capture artifacts, validation in diverse ICU settings	^66^^,^^67^

AI, Artificial intelligence; ARDS, Acute respiratory distress syndrome; AUC/AUROC, Area under the (receiver operating characteristic) curve; CDSS, Clinical decision support system; CNN, Convolutional neural network; COPD, Chronic obstructive pulmonary disease; COVID-19, Coronavirus disease 2019; CT, Computed tomography; DL, Deep learning; DSC, Dice similarity coefficient; EHR, Electronic health record; HRCT, High resolution computed tomography; ICU, Intensive care unit; ILD, Interstitial lung disease; IPF, Idiopathic pulmonary fibrosis; LSTM, Long short-term memory; ML, Machine learning; MRI, Magnetic resonance imaging; NIP, Non-specific interstitial pneumonia; NIV, Noninvasive ventilation; NLP, Natural language processing; NSIP, Non-specific interstitial pneumonia; OARs, Organs-at-risk; PET-CT, Positron emission tomography-computed tomography; PFT, Pulmonary function test; QA, Quality assurance; RCT, Randomized controlled trial; RT, Radiation therapy; SOFA, Sequential organ failure assessment; SVM, Support vector machine; XAI, Explainable artificial intelligence.

**Table 4:** Representative AI applications in radiation therapy and surgical robotics for lung diseases.

AI technology/ platform	Clinical application	Lung disease context	AI model/approach	Dataset/source	Performance/outcomes	Clinical validation/status	Reference(s)
U-Net–based Auto-Contouring	Tumor/OAR segmentation for RT planning	NSCLC, SCLC, metastatic lung CA	Deep CNN (U-Net)	Multicenter annotated CT/MRI datasets	DSC ≥0.90; 80 % reduction in planning time	Prospective, multi-center trials	^70^
CBCT-AI Adaptive Radiotherapy	Daily plan adaptation, dose optimization	Post-operative non-small cell lung cancer	CNN, deformable registration	Prospective clinical imaging (CBCT)	A 15% increase in local control; 30 % reduction in grade ≥2 pneumonitis; a 25 % lower OAR dose	Multicenter RCT, clinical adoption	^71^
Radiomic/XAI Dose Planning	Individualized RT regimens, toxicity prediction	NSCLC, CPFE, lung metastases	Radiomics, SHAP/attention XAI	Imaging + EHR + multi-omics	A 15% increase in AUROC for NTCP *vs*. DVH-only models; a 20 % increase in personalized dose adjustments	Early clinical, regulatory review	^72^
AI-Augmented Robotic Surgery (da Vinci, etc.)	3D AR-guided, real-time tumor navigation	Malignant (lung CA), benign lesions, and empyema	DL, AR, 3D modelling, vision transformers	Pre-operative CT/PET, intraoperative video, AR overlays	A 25 % increase in margin negativity, a 20 % reduction in complications, and a 1.5-day decrease in LOS compared to VATS.	Clinical registry, pilot studies	^73^
Intra-operative AI Metastasis Detection	Rapid lymph node/tumor assessment	NSCLC, SCLC, rare tumors	DL classifier, hyperspectral imaging	Surgical imaging combined with histopathology	Sensitivity 93 %, specificity 88 % *vs*. frozen section, 20 min faster reporting	Feasibility and prospective clinical trials	^74^
AI Motion Analysis/Skill Assessment	Automated surgeon training, credentialing	Malignant and benign lung diseases requiring RATS, VATS, or open thoracotomy	ML on kinematics, force/energy feedback	Simulator, intra-operative motion datasets	30 % fewer critical errors; significantly faster proficiency acquisition	Multi-institutional pilot	^75^^,^^76^
Ensemble AI for RT QA	RT quality assurance and dosimetric error prediction	All lung RT	Predictive ML, anomaly detection	RT machine logs, clinical plans	95 % error flagging, reduced delays and unplanned interruptions	Implementation studies, retrospective	^77^^,^^78^

3D AR, Three-dimensional augmented reality; AI, Artificial intelligence; AUROC, Area under the receiver operating characteristic curve; CA, Cancer; CBCT, Cone-beam computed tomography; CNN, Convolutional neural network; CPFE, Combined pulmonary fibrosis and emphysema; CT, Computed tomography; DL, Deep learning; DSC, Dice similarity coefficient; DVH, Dose-volume histogram; EHR, Electronic health record; LLM, Large language model; LOS, Length of stay; ML, Machine learning; MRI, Magnetic resonance imaging; NSCLC/SCLC, Non-small cell lung cancer/small cell lung cancer; NTCP, Normal tissue complication probability; OAR, Organs-at-risk; PET, Positron emission tomography; QA, Quality assurance; RATS, Robot-assisted thoracoscopic surgery; RCT, Randomized controlled trial; RT, Radiation therapy; SHAP, Shapley additive explanations; VATS, Video-assisted thoracoscopic surgery; XAI, Explainable artificial intelligence.

### Precision oncology and interventional decision support

In the management of thoracic malignancies, AI is redefining systemic therapy selection through the integration of cutting-edge computational frameworks and next-generation therapeutic modalities. The current frontier is characterized by the convergence of multimodal foundation models and medical LLMs with precision strategies including targeted therapy, immunotherapy, and their synergistic combinations.

For targeted therapy, mature CDSS platforms such as Tempus, OncoKB, and IBM Watson have transitioned from simple mutation cataloging to sophisticated genomic-phenotypic integration. Leveraging transformer-based architectures, these systems synthesize vast multi-omics data—including TKI-sensitive mutations (e.g., epidermal growth factor receptor [EGFR], anaplastic lymphoma kinase [ALK]) and sub-visual radiomic features—to predict drug resistance patterns before they manifest clinically. By cross-referencing real-time clinical trial databases, these platforms assist molecular tumor boards in selecting the most effective targeted agents with up to 80 % concordance with expert consensus^57^ (Table 3).

In the realm of immunotherapy, AI is addressing the critical challenge of identifying “responders” to immune checkpoint inhibitors (ICIs). Emerging multimodal LLMs and vision-language models (e.g., LLaVA-Med) are now capable of aligning programmed death-ligand 1 (PD-L1) expression levels, tumor mutational burden (TMB), and the spatial architecture of the tumor microenvironment (TME) derived from digital pathology. These models provide generative reasoning to forecast response to anti-programmed cell death protein 1 (PD-1)/PD-L1 therapies, moving beyond single-biomarker predictions. Furthermore, AI is instrumental in optimizing combined treatment strategies, such as chemo-immunotherapy or the sequential use of targeted agents and ICIs. By analyzing longitudinal “digital twin” simulations, AI helps clinicians balance the synergistic efficacy of combination therapies against the increased risk of immune-related adverse events (irAEs), ensuring a truly individualized precision oncology approach.^58^

Parallel to systemic therapy, AI has revolutionized localized treatments, shifting procedures from static interventions to dynamic, real-time adaptive therapies (Table 4). In radiation oncology, DL architectures such as U-Net and Transformers utilized by platforms like Qure.ai, RayStation, and Varian Ethos automate tumor and organ-at-risk (OAR) segmentation. These auto-contouring systems achieve greater than 90 % accuracy compared to expert consensus, drastically reducing time-to-plan by up to 80 %.^36^^,^^59^ Beyond static spatial planning, the integration of AI into online cone-beam CT (CBCT) adaptive radiotherapy enables daily plan modifications in response to anatomical changes. Multicenter randomized controlled trials demonstrate that this adaptability increases local control by 15 % while simultaneously reducing grade ≥2 radiation-induced pneumonitis by 30 % and decreasing overall OAR radiation dosing by 25 %. Furthermore, AI-guided dose optimization utilizes radiomics and explainable AI (XAI) frameworks like SHapley Additive exPlanations (SHAP) to forecast normal tissue complication probability (NTCP), allowing for highly individualized regimen tailoring. To ensure patient safety in these complex workflows, predictive ML models are deployed for routine quality assurance (QA), flagging dosimetric anomalies and machine log errors with 95 % accuracy to prevent unplanned treatment interruptions.^68^

In the surgical domain, the marriage of AI and robotic-assisted thoracic surgery (*e.g.,* the da Vinci system) has established unprecedented benchmarks for oncologic safety. AI-augmented consoles utilize deep learning 3D reconstructions and vision transformers to project augmented reality (AR) overlays of real-time tumor boundaries directly into the surgeon’s visual field. This enhanced spatial intelligence has led to profound clinical improvements, reducing positive margin rates from 15 % in conventional video-assisted thoracoscopic surgery (VATS) to merely 5 % in AI-assisted procedures, alongside a 20 % decrease in perioperative complications and a 1.5-day reduction in hospital length-of-stay. Moreover, AI provides critical intraoperative decision support through DL classifiers analyzing hyperspectral imaging to rapidly detect occult nodal metastases *in vivo*, achieving a 93 % sensitivity and an 88 % specificity approximately 20 minutes faster than traditional frozen sections.^69^ Finally, AI-powered kinematic motion analysis provides automated assessments of surgeon dexterity based on force feedback, correlating with a 30 % reduction in critical surgical errors during thoracic training (Table 4).

### Proactive management of chronic airway diseases

For chronic conditions such as COPD and asthma, the focus of CDSS has decisively shifted from reactive diagnostic identification to proactive management and exacerbation forecasting. Traditional machine learning models have now been augmented by multimodal LLMs and generative AI platforms capable of continuous, longitudinal monitoring. These advanced systems utilize vision-language transformers to integrate high-dimensional imaging with unstructured clinical narratives and real-time sensor data from wearables. For instance, in asthma management, AI-driven tools support early intervention and biologic therapy selection, achieving an AUROC of 0.89–0.93 for exacerbation prediction. In pilot randomized trials, these platforms significantly improved asthma control, evidenced by a 3–5 point increase in Asthma Control Test (ACT) scores and a 30 % reduction in emergency visits, even while navigating challenges in pediatric data harmonization.^60^^,^^61^ Similarly, for COPD, emerging generalist medical AI (GMAI) frameworks can synthesize acoustic cough analysis, daily spirometry, and EHR data to forecast exacerbations up to 30 days in advance. By acting as “clinical co-pilots”, these generative models provide explainable prognostic rationales, allowing clinicians to preemptively adjust therapies and enhance patient adherence through interactive, natural-language interfaces.^62^^,^^63^

### Rare fibrotic diseases and acute critical care

A critical frontier for AI is the management of rare and fibrotic lung diseases, where expert clinical guidelines are often scarce. In IPF and other ILDs, AI-assisted risk prediction tools such as ILD-RiskNet and LUNGOMIC-AI combine quantitative HRCT radiomics with multi-omics and medication adherence data. Utilizing support vector machines (SVM) and multimodal fusion, these models achieve an AUROC greater than 0.85 in predicting rapid disease progression.^79^ This effectively guides the timing of antifibrotic therapy adjustments and lung transplantation referrals—tasks where AI frequently outperforms traditional clinical scoring systems.^64^^,^^65^ Furthermore, in acute intensive care settings, triage tools rapidly developed during the pandemic have matured into robust platforms like ARDS-CDSS and COVID-TriageAI. By continuously processing bedside monitoring data, imaging, and EHR using real-time DL analytics, these systems predict the onset of acute respiratory distress syndrome (ARDS) and optimize mechanical ventilator management, yielding up to a 20 % relative improvement in AUROC for mortality prediction compared to traditional sequential organ failure assessment (SOFA) scores, thereby enabling more precise triage and timely escalation of care in ICU settings.^66^^,^^67^ Despite these profound translational advances across all therapeutic domains, persistent sociotechnical barriers—including the bioinformatic harmonization of heterogeneous data sources, hardware device dependency, and ongoing regulatory and reimbursement uncertainties—must be addressed to ensure the safe, equitable, and widespread clinical implementation of AI-powered respiratory care.

## AI in patient management and prognosis

Artificial intelligence is rapidly reshaping the landscape of patient management and prognostic assessment in pulmonary medicine, effectively transitioning the field from reactive, episodic care to proactive, data-driven disease management. The seamless integration of large-scale clinical, imaging, and molecular datasets—ranging from high-dimensional radiomics and genomics to real-world evidence from EHRs—has enabled the development of robust algorithms that support dynamic risk stratification across the full spectrum of respiratory diseases. A fundamental evolution in clinical prognostication involves the shift from traditional, linear statistical methods and early DL architectures to multimodal foundation models and generative AI. Unlike classical models that necessitate manual feature selection or early CNNs that function as “black boxes”, advanced frameworks—including medical large language models (Med-LLMs) and vision-language transformers—are capable of performing complex cross-modal reasoning. These systems autonomously synthesize latent prognostic factors from longitudinal clinical trajectories and unstructured physician notes, providing not only numerical risk scores but also explainable prognostic rationales. This transition enables highly dynamic, individualized risk estimations that adapt to a patient’s evolving clinical status in real-time. To systematically map this transition, Table 5 outlines the representative AI solutions ranging from established deep survival models to emerging multimodal predictive platforms—currently defining the benchmarks for prognostic modeling and remote patient management.

**Table 5:** Representative AI solutions for prognostic modelling and patient management in lung diseases.

AI solution	Application	AI model used	Dataset used	Performance benchmark	Clinical validation status	Limitations/ Challenges	Reference(s)
DeepSurv-Lung	Survival prediction in lung cancer	Deep neural network	Multi-omics + imaging data	C-index > 0.80; superior discriminatory power over TNM staging	Internal: Multi-omics training cohorts; external: validated in US and Asia	Needs prospective trials	^80^
AIPredict-ILD	Risk stratification in IPF	Ensemble ML, XGBoost, CNN	HRCT, spirometry, genomics	AUROC > 0.85 for 1-year progression; outperforms GAP score by ∼15 % in accuracy.	Multi-center retrospective	Generalizability, missing data	^81^^,^^82^
COPD Exacerbation Predictor	COPD exacerbation forecasting	RF, temporal CNN	EHR, remote monitoring, wearables	AUROC 0.85 for 30-day risk; precision-recall (PR) AUC ∼0.78.	Pilot implementation	Data heterogeneity, alert fatigue	^83^^,^^84^
Explainable Radiomics (SHAP-Lung)	Prognostic imaging biomarker interpretation	Radiomics + XAI tools	CT, PET-CT, MRI	Mean |SHAP value| > 0.15 for spiculation; identifies sub-visual prognostic biomarkers.	Research/clinical validation	Standardization of imaging	^87^^,^^88^
TeleCare-AI Lung	Remote monitoring and patient management	AI-enabled mHealth + NLP	Patient-reported + wearable data	A 30 % reduction in 30-day readmissions; sensitivity > 0.90 for symptom alerts.	Ongoing RCTs	Integration with EMRs	^85^^,^^86^

AI, Artificial intelligence; AUROC, Area under the receiver operating characteristic curve; C-index, Concordance index (a measure of predictive accuracy for survival models); CNN, Convolutional neural network; COPD, Chronic obstructive pulmonary disease; CT, Computed tomography; EHR/EMR, Electronic health record/electronic medical record; GAP score, Gender-age-physiology score; HRCT, High-resolution computed tomography; IPF, Idiopathic pulmonary fibrosis; mHealth, Mobile health; ML, Machine learning; MRI, Magnetic resonance imaging; NLP, Natural language processing; PET-CT, Positron emission tomography-computed tomography; RCT, Randomized controlled trial; RF, Random forest; SHAP, Shapley additive explanations; TNM, Tumor-node-metastasis; XAI, Explainable artificial intelligence; XGBoost, Extreme gradient boosting.

### Prognostic modeling

In the realm of thoracic oncology, AI has vastly superseded traditional anatomical staging frameworks by transitioning from narrow DL architectures to multimodal foundation models. While state-of-the-art prognostic models like DeepSurv-Lung (Table 5), established the baseline by integrating genomic mutations, radiomic features, and histopathological data to achieve a concordance index (C-index) greater than 0.80—significantly outperforming the TNM (Tumor, Node, Metastasis) staging system’s typical C-index of 0.60–0.70—the frontier has now shifted toward generative AI and LLMs.^80^

Emerging GMAI frameworks are now capable of performing cross-modal reasoning, aligning unstructured clinical narratives from electronic health records (EHR) with high-dimensional imaging data. Unlike earlier models that required manual feature engineering, these vision-language models (*e.g.,* LLaVA-Med) can interpret complex spatial tumor environments and generate explainable prognostic rationales, bridging the gap between numerical risk scores and clinical decision-making.

Similarly, AI is bridging critical prognostic gaps in rare and fibrotic lung diseases. In IPF, AI platforms like AIPredict-ILD leverage ensemble ML and CNNs to achieve an AUROC greater than 0.85 for 1-year mortality predictions, outperforming the traditional GAP (Gender, Age, and Physiology) score by an estimated 15 % in predictive accuracy.^81^^,^^82^ In cystic fibrosis (CF), DL models identify sub-visual structural biomarkers—such as subtle airway wall thickening—enabling the early detection of forced expiratory volume in one second (FEV_1_) decline months in advance.

The integration of medical LLMs into these workflows allows for the pre-emptive, personalized titration of cystic fibrosis transmembrane conductance regulator (CFTR) modulator therapies. By synthesizing real-time sensor data with longitudinal omics, these generative systems act as “clinical co-pilots”, ensuring intervention occurs before irreversible airway damage manifests.

### Remote monitoring and digital twins

For COPD and severe asthma, the prognostic focus extends beyond the clinic and into the outpatient environment through comprehensive digital health integration. The role of telemedicine has matured from simple symptomatic telemonitoring to the high-level integration of real-time physiological replicas, conceptually known as “digital twins”. A digital twin represents a virtual, patient-specific computational copy of the human respiratory system that is continuously updated with dynamic data from wearable biosensors, digital spirometry, and environmental sensors. This paradigm shift serves as the technological foundation for the “Hospital-at-Home” model, where the real-time AI analysis of subtle physiological deviations—such as variations in nocturnal desaturation, respiratory rate, and acoustic cough frequency—provides early detection of clinical decompensation before acute symptoms manifest.

AI-driven clustering techniques applied to longitudinal EHRs and wearable biosensor data now actively support the identification of high-risk “frequent exacerbator” phenotypes. Advanced prognostic platforms, such as the COPD Exacerbation Predictor, utilize random forests and temporal CNNs to forecast acute exacerbation events up to 30 days in advance. Achieving an impressive AUROC of 0.85 for 30-day risk, this system facilitates vital early pharmacological interventions, such as adjusting inhaled corticosteroid dosages, though pilot implementations note the critical need to mitigate false-positive alerts.^83^^,^^84^ Furthermore, holistic mobile health (mHealth) ecosystems like TeleCare-AI Lung leverage NLP to continuously analyze patient-reported outcomes alongside remote monitoring metrics. Ongoing randomized controlled trials (RCTs) suggest that these AI-enabled platforms significantly reduce 30-day hospital readmission rates, fundamentally decentralizing respiratory care and reducing the economic burden on acute healthcare facilities.^85^^,^^86^

### Explainability and translational bottlenecks

Despite these profound prognostic capabilities, translating complex predictive algorithms from *in silico* validation to bedside clinical decision-making faces critical barriers. The intrinsic “black box” nature of deep survival models often breeds clinical skepticism, as physicians cannot verify the rationale behind a high-risk prediction. To mitigate this trust deficit, the integration of XAI tools—such as explainable radiomics (SHAP-Lung)—has become essential. By visually and mathematically quantifying exactly how specific features (e.g., perinodular spiculation in lung cancer or regional emphysema distribution in COPD) contribute to a patient’s prognostic output, SHAP algorithms restore physician interpretability.^85^^,^^86^

However, as highlighted in the limitations of Table 5, widespread clinical adoption is continually challenged by institutional data heterogeneity and the lack of seamless integration with existing electronic medical records (EMRs). Furthermore, poorly calibrated remote monitoring platforms pose a significant risk of clinical “alert fatigue”, where physicians are overwhelmed by trivial notifications. Overcoming these bottlenecks requires large-scale, prospective validation trials across socioeconomically and demographically diverse cohorts to ensure that these AI prognostic models do not inadvertently perpetuate existing healthcare disparities.

## AI in molecular medicine

**Fig. 4:**
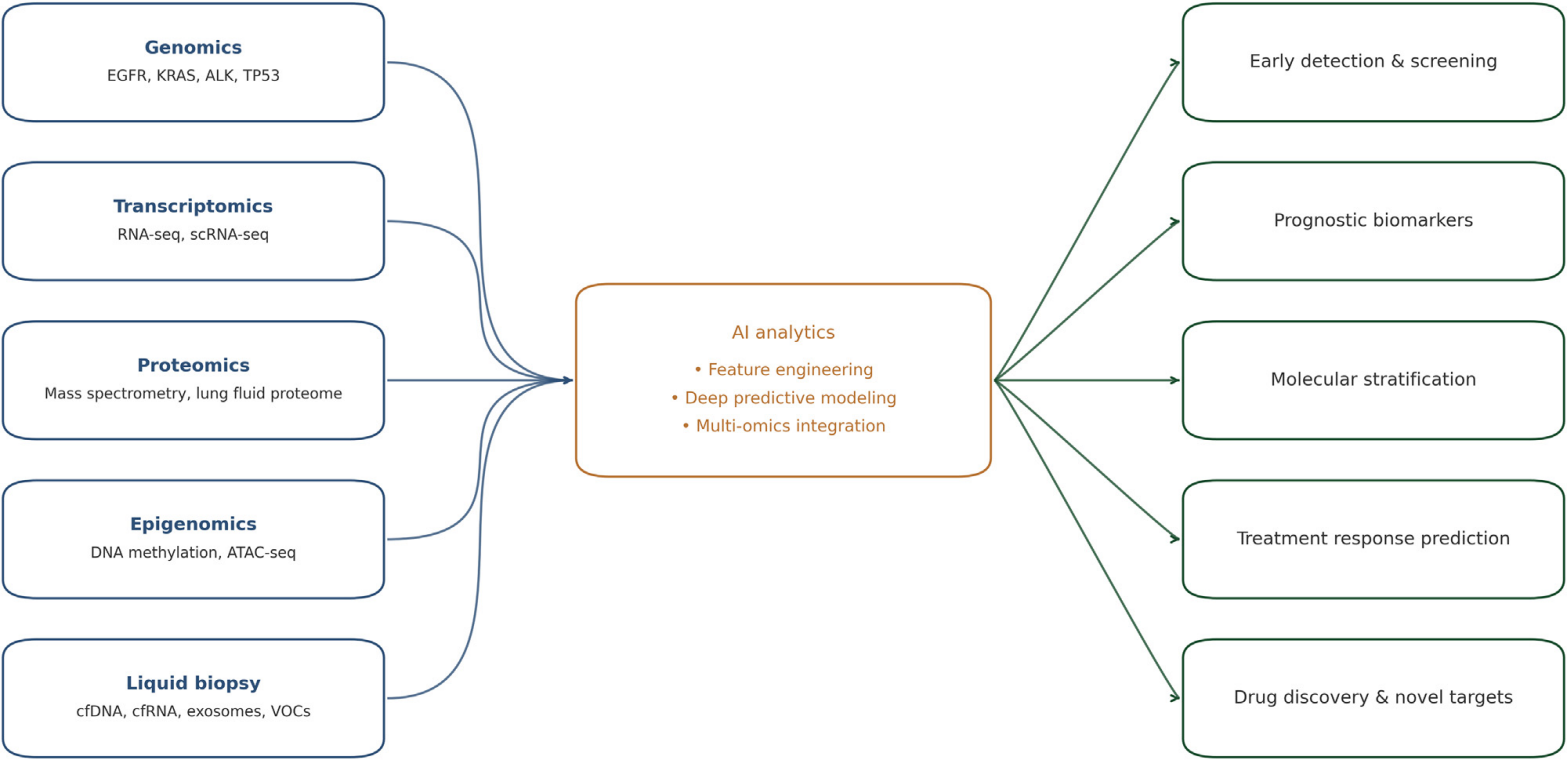
AI-enabled integration of multi-omics data for biomarker discovery in lung diseases. AI approaches are used to analyze large-scale datasets spanning genomics, transcriptomics, proteomics, epigenomics, and liquid biopsy assays (including circulating cfDNA, cfRNA, and VOCs). By combining these molecular data with imaging and clinical records, AI models uncover disease-specific biomarkers, support early detection, refine prognostic stratification, and inform therapeutic targeting in conditions such as lung cancer, pulmonary fibrosis, and COPD. AI, Artificial intelligence; ALK, Anaplastic lymphoma kinase; ATAC-seq, Assay for transposase-accessible chromatin using sequencing; cfDNA, Cell-free DNA; cfRNA, Cell-free RNA; COPD, Chronic obstructive pulmonary disease; EGFR, Epidermal growth factor receptor; KRAS, Kirsten rat sarcoma viral oncogene homolog; RNA-seq, RNA sequencing; scRNA-seq, Single-cell RNA sequencing; TP53, Tumor protein p53; VOCs, Volatile organic compounds.

Moving from macroscopic clinical management to the elucidation of underlying microscopic mechanisms, AI is increasingly critical in translating high-throughput molecular data into clinically actionable insights. By processing vast, multidimensional datasets, AI enables a fundamental shift toward biologically informed, individualized management across the entire spectrum of respiratory diseases. As conceptualized in Fig. 4, the contemporary AI-driven precision medicine workflow operates through a convergent pipeline: integrating genomic mutations, transcriptomic profiles, proteomics, epigenomics, and non-invasive liquid biopsies. Instead of relying on manual interpretation, central AI computational engines utilize advanced feature engineering and deep predictive modeling to map complex biological interactions, ultimately yielding precise clinical deliverables such as novel biomarker discovery, molecular stratification, and accelerated drug repurposing.

### Multi-omics and biomarker discovery

Beyond medical imaging, AI is extensively applied to integrated multi-omics datasets—encompassing genomics, transcriptomics, proteomics, and epigenomics—to uncover novel diagnostic and prognostic signatures. The rapid advancement of high-throughput molecular profiling has generated comprehensive datasets that elucidate the genetic and proteomic foundations of malignant, fibrotic, and inflammatory pulmonary conditions.

The critical role of AI in synthesizing these complex data streams for biomarker discovery is comprehensively delineated in Fig. 4. As illustrated, the contemporary AI-driven multi-omics workflow operates through a convergent, tripartite pipeline: (1) Multimodal inputs: The framework captures a holistic biological snapshot by ingesting distinct data layers, including genomics (*e.g., EGFR, KRAS, TP53* mutations), transcriptomics (bulk and single-cell RNA-seq), proteomics (mass spectrometry of lung fluid), epigenomics (DNA methylation and assay for transposase-accessible chromatin with high throughput sequencing [ATAC-seq]), and non-invasive liquid biopsies (cell-free DNA [cfDNA], cell-free RNA [cfRNA], and volatile organic compounds [VOCs]). (2) AI analytics engine: These heterogeneous inputs are processed by a central computational core. Instead of relying on manual interpretation, this engine utilizes advanced feature engineering, deep predictive modeling, and multi-omics integration protocols to map complex biological interactions. (3) Translational outputs: By combining these molecular signatures with clinical records, the AI models yield precise clinical deliverables: early detection, the identification of prognostic biomarkers, refined molecular stratification, treatment response prediction, and the discovery of novel drug targets for conditions like lung cancer and COPD.

A persistent mathematical bottleneck in molecular profiling is the “curse of dimensionality”. This challenge arises because the volume of genomic and proteomic features (p) frequently exceeds the number of available patient samples (n). In this p≫n paradigm, traditional statistical techniques often struggle with the extreme heterogeneity, sparsity, and noise inherent in the data. In contrast, advanced AI methodologies—including DL, SVM, and ensemble methods like RF—are uniquely capable of mining high-dimensional spaces to identify complex, non-linear biological associations. Table 6 provides a comprehensive overview of these foundational AI tools and their specific clinical applications.

**Table 6:** AI applications in multi-omics and precision medicine for lung diseases.

AI Tool/ platform	Primary application	Core AI model(s)	Datasets	Benchmark	Clinical validation	Limitations/challenges	Data type/modality	Regulatory/clinical Status	Reference(s)
**DeepVariant (Google)**	NGS variant calling	Deep CNN	1000 Genomes, GIAB	Sensitivity/PPV >99 %; outperforms standard variant callers	Research/clinical use	High computational demand, NGS platform bias	Genomic sequencing	Research/clinical lab use only	^89^^,^^90^
**AlphaFold**	Protein structure prediction (proteomics)	Transformer-based DL	PDB	Near-experimental RMSD accuracy (<2 Å for many targets)	Validated (research)	Limited to known sequences, interpretability	Proteomics	Pharmaceutical research and development	^91^
**IBM Watson Oncology**	Precision oncology decision support	NLP, ML, ensemble	Large clinical, genomic datasets	∼80 % concordance with expert molecular tumor boards	Some hospitals	Explainability, variable generalizability	Clinical + genomic data	Some FDA clearance	^92^^,^^93^
**LungGen DL**	Lung cancer subtype classification	CNN, autoencoder	TCGA-LUAD, GEO	∼89 % accuracy, AUROC >0.9	Preclinical	Generalizability, population differences	Transcriptomic, mutation data	Research only	^94^^,^^95^
**AI-LB Lung**	Cancer detection and monitoring using liquid biopsy	RF, ensemble learning	cfDNA/ctDNA, cfRNA, clinical data	Sensitivity 94 %, specificity 91 % (pilot)	Clinical trials	Sample standardization, cohort size	Liquid biopsy, cfDNA/cfRNA	In trials	^94^^,^^95^
**PRS-Lung**	Polygenic risk for lung cancer	AI-based polygenic	UK Biobank, GWAS	AUC ∼0.72 for NSCLC	Early implementation	Rare variant limitation, population bias	Genomic risk profiling	Genetic counselling	^96^^,^^97^
**LUNGOMIC AI**	IPF multi-omics subtyping	Multi-kernel SVM, AE	Bulk and single-cell multi-omics (RNA-seq, ATAC-seq)	85.7 % subtype prediction accuracy	Research phase	High dimensionality, overfitting risk	Transcriptomics, epigenomics	Research only	^98^^,^^99^
**GENEVA**	Asthma exacerbation biomarker	Random forest	Cytokine, gene expression	87 % accuracy, small sample validated	Preclinical	Sample size, external validation needed	Multi-modal biomarkers	Not in clinical use	^100^^,^^101^
**cfRNA-COVID Lung Risk**	COVID-19 severity risk prediction	DL	cfRNA-Seq, clinical data	Improved early triage, pilot stratification	Emergency use	Sampling timing, cohort heterogeneity	cfRNA	Limited EUA, pilot clinics	^102^^,^^103^

AE, Autoencoder; AI: Aritifical intelligence; ARDS, Acute respiratory distress syndrome; ATAC-seq, Assay for transposase-accessible chromatin using sequencing; AUC/AUROC, Area under the (receiver operating characteristic) curve; cfDNA/ctDNA, Cell-free DNA/circulating tumor DNA; cfRNA, Cell-free RNA; CNN, Convolutional neural network; COVID-19, Coronavirus diseases 2019; DL, Deep learning; EUA, Emergency use authorization; FDA, Food and Drug Administration; GEO, Gene expression omnibus; GIAB, Genome in a Bottle; GWAS, Genome-wide association study; IPF, Idiopathic pulmonary fibrosis; ML, Machine learning; NGS, Next-generation sequencing; NLP, Natural language processing; NSCLC, Non-small cell lung cancer; PDB, Protein data bank; PPV, Positive predictive value (precision); RF, Random forest; RMSD, Root-mean-square deviation (of atomic positions); RNA-seq, RNA sequencing; SVM, Support vector machine; TCGA-LUAD, The cancer genome atlas lung adenocarcinoma.

In thoracic oncology, this integrative analytical capacity allows for superior patient stratification. For instance, supervised neural networks can distinguish between adenocarcinoma and squamous cell carcinoma using complex datasets like The Cancer Genome Atlas Lung Adenocarcinoma (TCGA-LUAD) with unprecedented precision. Furthermore, tools like DeepVariant (Table 6) have markedly enhanced the accuracy of variant calling in next-generation sequencing (NGS) by treating genomic detection as an image recognition task, significantly reducing false positives in low-frequency somatic mutations. Moving beyond single-gene paradigms, AI-driven polygenic risk scoring (PRS) models now seamlessly integrate genome-wide association study (GWAS) data with clinical variables to estimate individual susceptibility more effectively than traditional unimodal risk models.

Crucially, AI’s ability to delineate cryptic disease subtypes is proving transformative not only for rare and chronic conditions but also for acute infectious lung diseases, most notably COVID-19, where experimental data are inherently limited. In CF, revolutionary AI models like AlphaFold (Table 6) are utilized to predict three-dimensional protein structural changes, facilitating the rational design of novel CFTR modulators. In IPF, the unsupervised ML clustering of single-cell RNA sequencing (scRNA-seq) and spatial transcriptomics has revealed novel, aggressive molecular endotypes associated with rapid fibrotic progression, providing a critical window for earlier targeted intervention. Similarly, for complex airway diseases like asthma and COPD, ML algorithms applied to transcriptomic profiles have successfully identified distinct inflammatory endotypes (*e.g.,* Th2-high *vs*. Th2-low), precisely guiding the selection of targeted biologic therapies. During the COVID-19 pandemic, this molecular synthesis allowed DL classifiers to predict ARDS severity directly from plasma cfRNA signatures, optimizing intensive care resource allocation.

### Liquid biopsies

Parallel to tissue-based multi-omics, an unprecedented convergence of AI and liquid biopsy profiling is fundamentally reshaping the landscape of early diagnosis and dynamic therapeutic monitoring (Table 7). Moving beyond the limitations of invasive tissue biopsies, deep neural networks and ensemble models now enable the rapid extraction of high-dimensional biological information from plasma, bronchoalveolar lavage (BAL), and exhaled breath. In thoracic oncology, multi-cancer early detection models, such as Galleri, utilize ML to decode complex cell-free DNA (cfDNA) methylation patterns. These models achieve sensitivities exceeding 95 % and specificities over 99 %, dramatically accelerating the time to diagnosis and facilitating routine minimal residual disease (MRD) monitoring.^104^

**Table 7:** AI-powered liquid biopsy and multi-omics diagnostics across lung diseases.

Application area	Disease focus	Sample type(s)	AI approach	Clinical use/impact	Benchmark/ performance	Limitations/ challenges	Reference(s)
Early detection, risk stratification	Lung cancer	Plasma (cfDNA/ctDNA, cfRNA, exosomes, CTCs)	CNNs, DNNs, SVM, ensemble models	Mutation detection, therapy selection, MRD monitoring; reduces need for invasive biopsy	Sensitivity >95 %, specificity >99 % (*e.g.,* Galleri)	Data heterogeneity, reimbursement	^105^
Subtyping, progression prediction	IPF, ILD	Plasma (cfDNA, cfRNA, proteome), BAL	Multi-kernel SVM, random forest, deep learning	Non-invasive subtype differentiation, antifibrotic response prediction, trial enrolment	Accuracy >85 % (*e.g.,* LUNGOMIC AI)	Sample standardization, model generalizability	^106^^,^^107^
Exacerbation risk, phenotyping	COPD	Plasma, serum, sputum (miRNA, proteins, methylome)	Random forest, LSTM, multimodal fusion	Predicts rapid FEV_1_ decline, stratifies frequent exacerbators, guides therapy	AUC 0.80–0.88 (COPDGene, SPIROMICS)	EHR data harmonization	^108^
Endotype discovery, early intervention	Asthma (adult, paediatric)	Blood, breath (cytokines, miRNA, VOCs)	XGBoost, CNN, explainable AI	Identifies severe/Th2-high endotypes, forecasts exacerbations, personalizes biologics	AUC 0.89–0.93 (GENEVA, PRS-Lung)	Pediatric sample bias	^62^
Early severity triage, dynamic risk	ARDS, COVID-19, sepsis	Plasma (cfRNA, proteome), blood	Deep learning, ensemble models	Predicts progression to respiratory failure, informs ICU allocation	AUROC >0.90 (cfRNA-COVID)	Validation in diverse settings	^109^^,^^110^
Rapid diagnosis, pathogen typing	Tuberculosis, pneumonia	Plasma, serum, sputum (cfDNA, protein)	Random forest, CNN	Non-invasive diagnosis, supports antibiotic stewardship, resource-limited use	Sensitivity/ specificity >90 %	Sample transport, endemic strain variation	^111^
Non-invasive differential diagnosis	Multiple (lung cancer, COPD, asthma, ILD)	Exhaled breath (VOCs)	CNN, SVM, multimodal fusion	Point-of-care screening, outpatient monitoring, phenotyping	Accuracy >90 % (VOC-AI)	Device calibration, environmental noise	^112^^,^^113^

AI, Artificial intelligence; ARDS, Acute respiratory distress syndrome; AUC/AUROC, Area under the (receiver operating characteristic) curve; BAL, Bronchoalveolar lavage; cfDNA/ctDNA, Cell-free DNA/circulating tumor DNA; cfRNA, Cell-free RNA; CNN, Convolutional neural network; COPD, Chronic obstructive pulmonary disease; COVID-19, Coronavirus disease 2019; CTC, Circulating tumor cell; DNN, Deep neural network; EHR, Electronic health record; FEV_1_, Forced expiratory volume in one second; ICU, Intensive care unit; ILD, Interstitial lung disease; IPF, Idiopathic pulmonary fibrosis; LSTM, Long short-term memory; miRNA, MicroRNA; MRD, Minimal residual disease; PRS, Polygenic risk score; RF, Random forest; SVM, Support vector machine; Th2, T helper 2; VOC, Volatile organic compound; XGBoost, Extreme gradient boosting.

Beyond malignancy, AI is unlocking the prognostic potential of liquid biopsies in chronic and acute respiratory conditions. In obstructive airway diseases, multimodal fusion models applied to large cohorts (e.g., COPDGene and SPIROMICS) analyze plasma microRNAs and methylomes to predict rapid FEV_1_ decline, effectively stratifying frequent exacerbators to guide proactive interventions. Similarly, in acute care settings, DL models applied to plasma cfRNA (cfRNA-COVID) have demonstrated the ability to predict progression to respiratory failure in ARDS with AUROCs exceeding 0.90, dynamically informing critical ICU resource allocation. Furthermore, the emerging frontier of “breathomics” represents the ultimate non-invasive diagnostic modality. AI models such as VOC-AI interpret complex VOC profiles in exhaled breath using CNNs and SVMs, accurately differentiating between lung cancer, COPD, and asthma with validated accuracies exceeding 90 %, entirely bypassing the need for blood draws.

### Drug discovery and repurposing

AI is fundamentally reshaping pulmonary pharmacology by overcoming the historical bottlenecks of traditional drug development—namely, prohibitive costs, protracted timelines, and high clinical attrition rates. As detailed in Table 8, AI platforms are radically accelerating both the *de novo* design of novel molecules and the strategic repurposing of existing therapeutics. For conditions historically limited by “undruggable” targets, AI is driving structural intelligence. At the foundational level, Transformer-based DL architectures like AlphaFold have achieved near-experimental accuracy in 3D protein structure prediction. Moving from structural prediction to molecular generation, platforms leveraging GANs and reinforcement learning have achieved unprecedented development speeds. Most notably, Insilico Medicine utilizing the generative tensorial reinforcement learning (GENTRL) model successfully designed, synthesized, and preclinically validated a novel pan-fibrotic discoidin domain receptor 1 (DDR1) kinase inhibitor for fibrotic diseases in merely 21 days—a fraction of the traditional multi-year timeline. Concurrently, generative models such as Chemistry42 and MolGPT are actively screening massive chemical libraries to identify highly selective kinase inhibitors for non-small cell lung cancer (NSCLC) and complex respiratory infections.

**Table 8:** AI-driven drug discovery and repurposing for lung diseases.

AI platform	Primary lung disease focus	AI model used	Dataset/source	Key findings/performance	Clinical validation status	Challenges	Reference(s)
RAlphaFold2	Lung cancer, CF, IPF, asthma	Transformer-based DL	Protein data bank, UniProt	High-accuracy structure prediction, enables rational drug design for undruggable targets	Pharmaceutical research and development, early translational	Computational cost; limited for disordered proteins	^114^
Atomwise	NSCLC, fibrotic ILD, COPD	CNN-based DL	ChEMBL, DrugBank, molecular libraries	Identified novel kinase inhibitors, anti-fibrotic and anti-inflammatory leads	Preclinical/early clinical	Requires experimental validation; target specificity	^115^
BenevolentAI	COVID-19 pneumonia, ILDs, COPD	NLP, graph neural networks	Biomedical literature, patents, omics	Baricitinib repurposed for severe COVID-19; candidate anti-fibrotics for ILD	FDA-approved for COVID-19; ongoing studies	Labelled data; generalizability	^116^^,^^117^
Insilico Medicine/ GENTRL	Lung cancer, ILDs, COPD	GANs, reinforcement learning, GENTRL	Chemical/genomic data, multi-omics	Rapid generation of novel anti-fibrotic, anti-cancer compounds	Preclinical; DDR1 inhibitor in 21 days	Real-world validation, dataset diversity	^118^
Schrödinger AI	NSCLC, PAH, asthma, infection	ML-based simulation, virtual screening	MD, DrugBank, ChEMBL	Improved virtual screening for kinase inhibitors, anti-infectives	Pharmaceutical research and development	Experimental validation, scalability	^119^
QPOP	Drug-resistant TB, COPD, asthma	Empirical phenotypic ML	Clinical isolates, patient-derived cells	Optimizes multi-drug combinations, personalized regimens for complex lung infections/disease	Clinical pilot studies	Complexity of empirical data; clinical trial need	^120^^,^^121^
DeepChem/ DeepDDS	Asthma, COPD, TB, lung cancer	Graph neural networks, DL, multi-omics	ChEMBL, PubChem, EHR, transcriptomics	Predicts synergistic drug pairs, anti-inflammatory leads for severe asthma, TB, NSCLC	Preclinical	Dataset bias, limited external validation	^122^
CANDO	ILD, PAH, infection, lung cancer	Proteochemometric ML, CNNs, RFs	FDA/EMA drugs, human proteome	Predicts multi-target efficacy, supports repurposing for rare and resistant lung diseases	Retrospective, research-stage	Prediction accuracy, need for prospective trials	^123^
BenevolentAI/ COVID-19	Severe viral pneumonia	NLP, GNN	Global COVID-19 clinical & molecular data	Baricitinib repurposed, inclusion in international guidelines	FDA/EMA/WHO-approved	Changing viral evolution, label noise	^124^^,^^125^
Chemistry42	NSCLC, advanced solid tumours	GANs, RNNs, transfer learning	Public/private chemical/biological data	CDK8 inhibitors for solid tumors, strong antiproliferative activity	Preclinical/ experimental	Generalizability, scalability	^126^^,^^127^
MolGPT, T5MolGe	NSCLC, ILD, infection	Transformer-based generative models	GuacaMol, ChEMBL, DrugBank	Novel kinase inhibitors; high-efficiency molecule generation for CFTR and EGFR targets	Research/proof-of-concept	Needs clinical validation, toxicity screening	^128^

AI, Artificial intelligence; CANDO, Computational analysis of novel drug opportunities; CDK8, Cyclin-dependent kinase 8; CF, Cystic fibrosis; CFTR, Cystic fibrosis transmembrane conductance regulator; ChEMBL, A chemically partitioned database of bioactive molecules; CNN, Convolutional neural network; COPD, Chronic obstructive pulmonary disease; COVID-19, Coronavirus disease 2019; DDR1, Discoidin domain receptor 1; DL, Deep learning; EGFR, Epidermal growth factor receptor; EHR, Electronic health record; EMA, European Medicines Agency; FDA, Food and Drug Administration; GANs, Generative adversarial networks; GENTRL, Generative tensorial reinforcement learning; GNN, Graph neural network; ILD, Interstitial lung disease; IPF, Idiopathic pulmonary fibrosis; MD, Molecular dynamics; ML, Machine learning; NLP, Natural language processing; NSCLC, Non-small cell lung cancer; PAH, Pulmonary arterial hypertension; QPOP, Quadratic phenotypic optimization platform; RF, Random forest; RL, Reinforcement learning; RNN, Recurrent neural network; TB, Tuberculosis; WHO, World Health Organization.

For rare and acute diseases where development timelines are critical, AI-driven drug repurposing yields immediate actionable insights. By deploying NLP and graph neural networks (GNNs) across vast biomedical literature and multi-omics databases, platforms map hidden pharmacological relationships. A landmark triumph of this knowledge graph approach occurred during the COVID-19 pandemic when the BenevolentAI platform rapidly identified the rheumatoid arthritis drug baricitinib as a potent anti-viral candidate for severe viral pneumonia, leading to its subsequent FDA approval and global clinical deployment. In the realm of highly resistant diseases, such as drug-resistant tuberculosis, platforms like QPOP and DeepChem utilize empirical phenotypic ML to model complex drug synergy, successfully predicting highly effective, personalized multidrug regimens that overcome acquired resistance.

Finally, for the continual optimization of these dynamic therapeutic strategies, researchers are increasingly utilizing reinforcement learning (RL) frameworks. As visually conceptualized in Supplementary Fig. 2, the RL paradigm functions through continuous clinical interaction: an AI agent observes the patient’s current state (e.g., longitudinal spirometry, multi-omics), executes a specific action (e.g., adjusting biologic therapies), and receives a mathematical reward based on physiological improvement. Through this iterative optimization, AI is poised to learn highly adaptive, personalized treatment pathways, solidifying its role not merely as a diagnostic tool, but as an indispensable architect of precision pulmonary care.

## Challenges and future directions

**Fig. 5:**
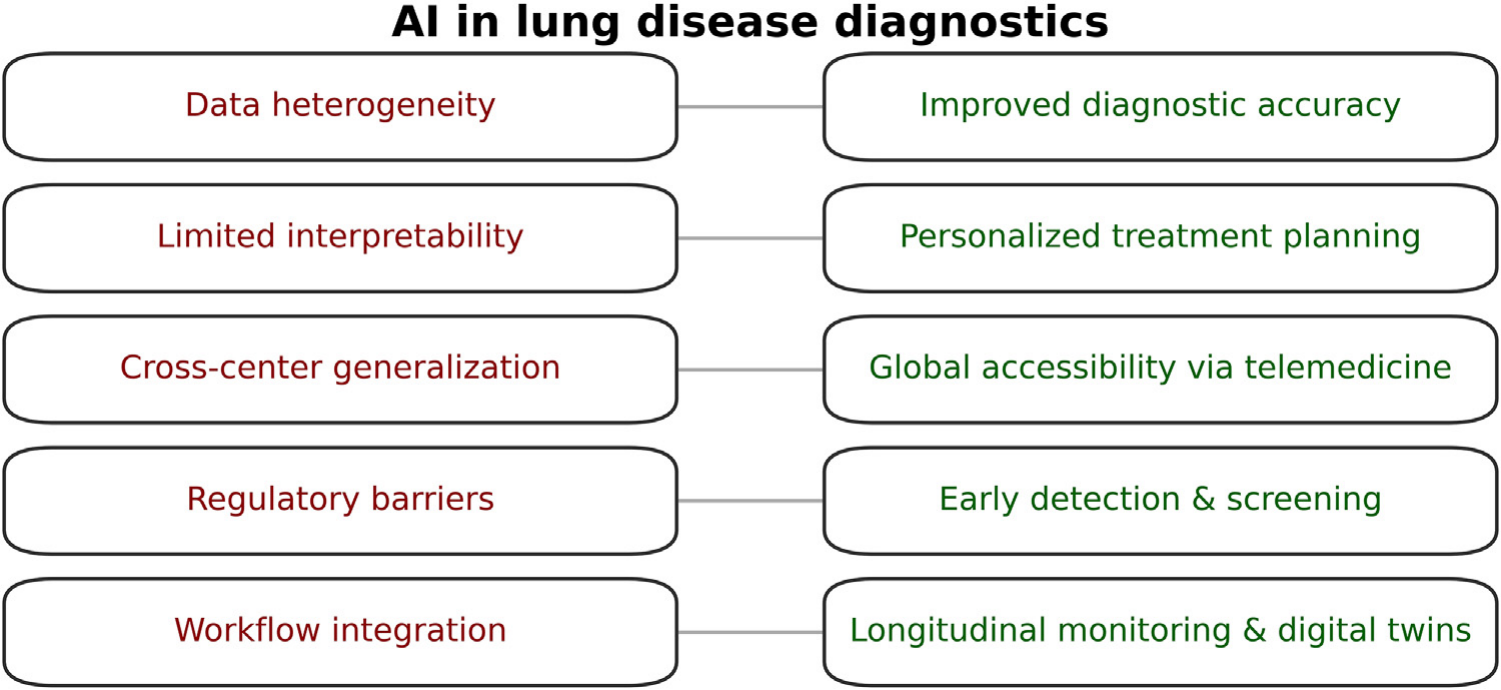
Challenges and opportunities for AI in lung disease diagnostics and management. Challenges include limited data standardization, privacy concerns, model interpretability, and regulatory approval barriers. Opportunities to overcome these obstacles include harmonized multicenter datasets, privacy-preserving machine learning (*e.g.,* federated learning), explainable AI frameworks, and integration into clinical decision support systems. Addressing these issues will be essential for the safe, equitable, and scalable implementation of AI in pulmonary care. AI, Artificial intelligence.

Despite the transformative promise of AI across the pulmonary care continuum, the path to widespread clinical adoption is beset by a profound “sociotechnical gap”—the fundamental disconnect between mathematically high-performing algorithms and their safe, equitable integration into complex human-centric healthcare environments. Transitioning from *in silico* proof-of-concept to standard clinical practice requires overcoming multifaceted barriers. As conceptualized in Fig. 5, these challenges—and their corresponding future solutions—span data integrity, model interpretability, and institutional governance, ultimately charting the roadmap toward integrated respiratory intelligence.

### Data and bias

A primary scientific bottleneck in medical AI is the lack of universal data standardization and the inherent “bias of representation”. Respiratory datasets—comprising multiscale pixel imaging, high-frequency wearable sensor outputs, and longitudinal spirometry—often exist in fragmented, institution-specific silos. Furthermore, major publicly available training cohorts frequently exhibit severe demographic skewing; for instance, algorithms trained predominantly on populations of European descent suffer significant performance degradation when applied to ethnic minorities or pediatric demographics. In rare respiratory conditions (e.g., pulmonary alveolar proteinosis or lymphangioleiomyomatosis), this data scarcity creates a vicious cycle where AI models are prone to overfitting, paradoxically marginalizing the orphan disease patients who could benefit most from precision medicine.

Equally critical is the pervasive “model drift” phenomenon—the stark drop in predictive accuracy when models transition from highly curated, retrospective training datasets to the noisy, variable realities of prospective clinical workflows (*e.g.,* varying CT scanner calibrations or slice thicknesses). To bridge this gap, future research must strictly adhere to specialized reporting guidelines, such as CONSORT-AI and SPIRIT-AI, ensuring that multi-center randomized controlled trials (RCTs) validate the prospective clinical utility, rather than merely the retrospective mathematical precision, of these algorithms.

### Interpretability and trust

The intrinsic “black box” nature of DL architectures creates a significant barrier to clinician “buy-in”. For a pulmonologist to confidently act upon an AI-generated high-risk alert for impending COPD exacerbation or rapid fibrotic progression, they must understand the physiological rationale driving the prediction. Without this transparency, uncalibrated AI systems risk inducing “alert fatigue”, overwhelming clinical workflows with unactionable notifications. Overcoming this trust deficit requires the universal implementation of XAI frameworks, such as SHAP (SHapley Additive exPlanations) or local interpretable model-agnostic explanations (LIME). By mathematically quantifying and visually highlighting the specific clinical or radiomic features (*e.g.,* nocturnal desaturation trends or perinodular vascularity) that influenced a model’s output, XAI transitions AI from an opaque oracle into a collaborative, verifiable diagnostic partner. Furthermore, modern medical education must evolve to foster “algorithmic literacy”, equipping the next generation of respiratory physicians to critically interpret AI outputs and identify subtle diagnostic drift.

### Regulatory and economic barriers

As algorithms mature toward semi-autonomous roles in high-stakes environments—such as adaptive radiotherapy planning or real-time surgical navigation—the most formidable barriers are institutional rather than technical. The deployment of DL creates a legal liability vacuum: current medicolegal frameworks are ill-equipped to apportion responsibility for an AI-induced medical error among software developers, hospital IT administrators, and the prescribing physician. Regulatory bodies are beginning to adapt, with initiatives like the FDA’s “Software as a Medical Device” (SaMD) precertification program attempting to regulate continuous-learning algorithms that evolve post-deployment.

However, the ultimate bottleneck to widespread clinical integration is economic sustainability. Unlike cardiology, which has established specific current procedural terminology (CPT) codes for AI-driven coronary analyses, respiratory AI currently lacks a standardized reimbursement framework. Without clear financial pathways for healthcare systems to recoup the “total cost of ownership”—encompassing high-performance graphics processing unit (GPU) infrastructure, cybersecurity maintenance, and continuous model auditing—advanced AI will remain a specialized luxury confined to well-funded academic centers, exacerbating global healthcare disparities.

### Future trends (FL, foundation models)

Looking forward, the next decade of pulmonary medicine will be defined by two transformative technological paradigms designed to overcome these historical bottlenecks: privacy-preserving AI and multimodal foundation models (MFMs).

To resolve the paradox between the need for massive datasets and strict data privacy regulations (e.g., Health Insurance Portability and Accountability Act [HIPAA], General Data Protection Regulation [GDPR]), the field is rapidly pivoting toward FL and swarm learning. These decentralized architectures allow AI models to be collaboratively trained across global institutional networks without ever exchanging raw, identifiable patient data. This guarantees data sovereignty while achieving the statistical power necessary for robust, unbiased model generalizability. Coupled with the use of GANs to create high-fidelity “synthetic data” for rare lung diseases, FL provides a secure pathway to democratize precision medicine.

Simultaneously, the architectural landscape is shifting from narrow, task-specific algorithms toward MFMs and GMAI. Unlike previous iterations confined to single modalities, next-generation LLMs (e.g., Med-PaLM) and vision-language models (VLMs, e.g., CLIP) project radiological pixels, histopathological slides, multi-omics profiles, and unstructured clinical narratives into a unified representational space. In the near future, these generative models will act as highly sophisticated “co-pilots” during MDT meetings—capable of zero-shot diagnostic reasoning for rare pathologies, automatically synthesizing decades of patient history, and cross-referencing real-time clinical trial literature to propose optimal, individualized therapeutic regimens.

Ultimately, the convergence of privacy-preserving federated networks and multimodal generative intelligence will catalyze the transition of respiratory medicine from a reactive discipline into a truly predictive, equitable, and globally scalable science.

## Conclusions

AI has fundamentally evolved from a promising adjunct to an indispensable pillar of contemporary pulmonary medicine. By integrating vast, heterogeneous biomedical data—spanning from pixel-level imaging and digital pathology to multi-omics profiles and real-time wearable sensor data—AI is transforming every stage of the respiratory care continuum. This review has critically synthesized the shift from task-specific automation to multimodal reasoning frameworks, highlighting how AI-enabled solutions are moving clinical decision-making away from subjective judgment toward highly accurate, data-driven precision medicine.

In clinical practice, the transformative power of AI is most evident in its ability to deliver personalized interventions: from radiologist-level nodule detection and automated tuberculosis screening in resource-limited settings to the identification of actionable molecular targets in rare lung diseases like CF and pulmonary hypertension. On the patient management front, the emergence of “digital twins” and AI-driven remote monitoring is catalyzing a shift toward a “Hospital-at-Home” model, enabling proactive intervention and improving long-term outcomes for chronic conditions like COPD and asthma. Furthermore, the rapid rise of LLMs and multimodal foundation models is redefining clinical workflows, providing clinicians with sophisticated reasoning engines capable of synthesizing complex longitudinal patient histories.

Nevertheless, achieving safe, equitable, and universal clinical adoption remains a multifaceted challenge. As this review has underscored, the “sociotechnical gap” between retrospective algorithmic accuracy and real-world prospective utility must be bridged through rigorous, multi-center randomized controlled trials. Beyond technical performance, the respiratory care community must navigate critical non-technical barriers, including the legal liability vacuum inherent in “black box” systems, the need for standardized pricing and reimbursement models, and the paramount importance of data privacy through solutions like FL.

Looking ahead, a multidisciplinary, collaborative ecosystem—uniting AI scientists, pulmonologists, radiologists, pathologists, ethicists, and policymakers—will be essential to establish fair and transparent AI standards. Medical education must likewise evolve, empowering the next generation of clinicians with the “algorithmic literacy” required for responsible AI adoption. Ultimately, the integration of generative AI, digital twins, and privacy-preserving collaborative models holds the potential to reshape respiratory medicine into a truly predictive, equitable, and outcome-oriented discipline, ensuring that the benefits of precision medicine reach all patients, regardless of disease rarity or geographic location.

## CRediT authorship contribution statement

**Zhe Chen:** Writing – review & editing, Writing – original draft, Visualization, Validation, Supervision, Software, Resources, Project administration, Methodology, Conceptualization. **Zhiyong Tang:** Writing – review & editing, Writing – original draft, Visualization, Validation, Methodology. **Rob M. Ewing:** Writing – review & editing, Writing – original draft, Supervision, Project administration. **Zehor Belkhatir:** Writing – review & editing, Writing – original draft, Supervision, Project administration. **Yihua Wang:** Writing – review & editing, Writing – original draft, Visualization, Validation, Supervision, Project administration.

## Declaration of competing interests

The authors declare that they have no known competing financial interests or personal relationships that could have appeared to influence the work reported in this paper.

## Appendix Supplementary materials

Image, application 1
