# Supplementary material for: Artificial intelligence in respiratory medicine: From diagnosis to treatment and future directions
Source: Chin Med J Pulm Crit Care Med. 2026 Jun 6;4(2):99–116. doi: 10.1016/j.pccm.2026.05.005 (PMC13323542; doi:10.1016/j.pccm.2026.05.005)
Supplement: Supplementary file 1 [file mmc1.docx]

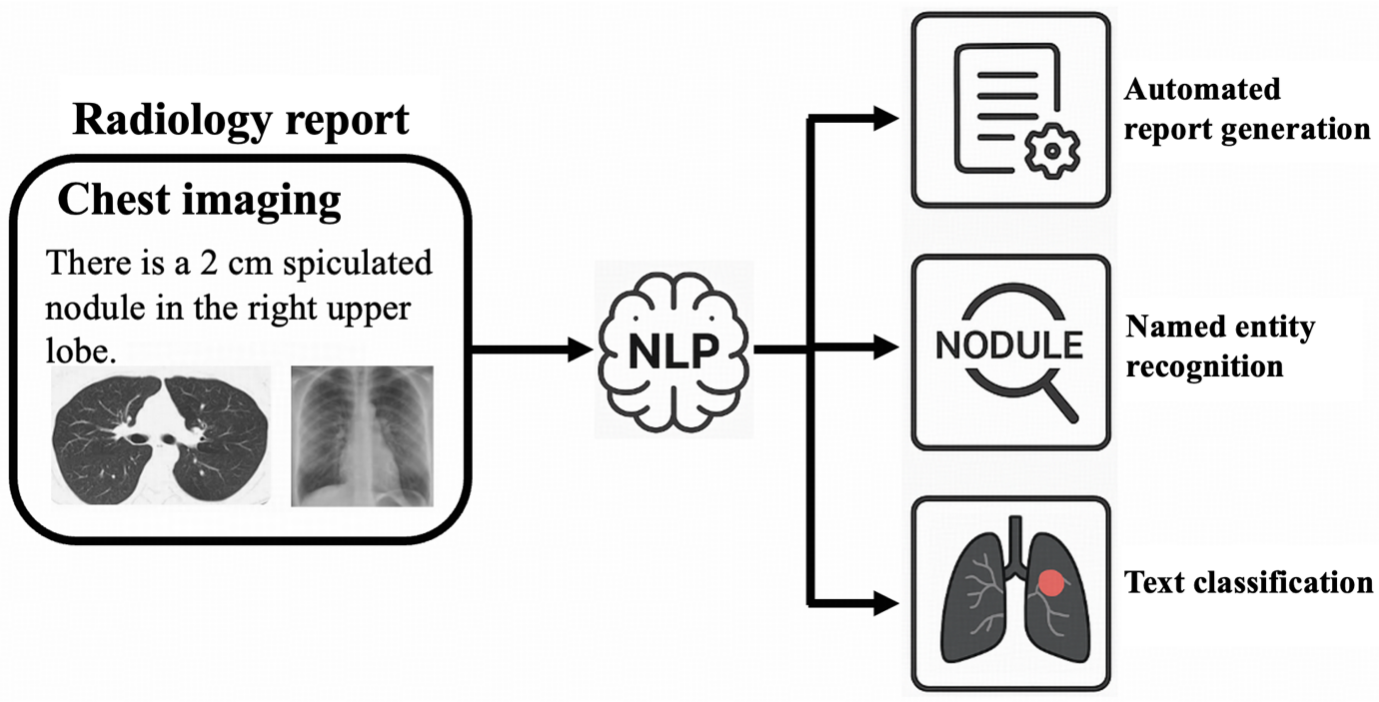


**Supplementary Fig. 1. Applications of natural language processing (NLP) in clinical radiology workflows.** A sample chest imaging report describing a 2-cm spiculated nodule is processed by NLP models. Downstream applications include automated report generation, named entity recognition (e.g., automated nodule detection and precise anatomical localization), and sophisticated text classification. By transforming unstructured clinical narratives into structured data, NLP facilitates large-scale data mining, predictive modelling, and seamless integration into clinical decision support systems.


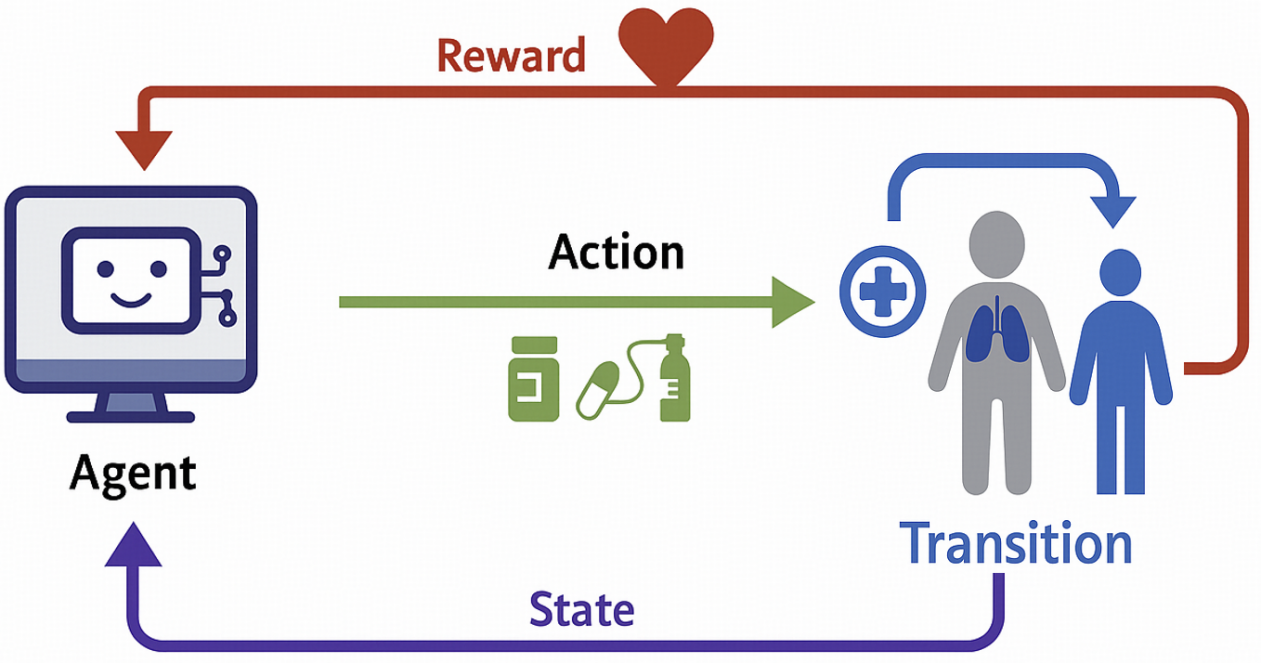


**Supplementary Fig. 2. Reinforcement learning framework for clinical decision-making in lung diseases.** In RL, an AI agent interacts with the patient environment by observing the current state (e.g., physiological parameters, imaging, clinical context) and taking actions (e.g., initiating or adjusting therapies). The environment then transitions to a new state reflecting patient response, and the agent receives a reward signal (e.g., improvement in clinical outcomes). Through iterative optimization, RL agents can learn adaptive, personalized treatment strategies for conditions such as COPD, asthma, and lung cancer, with the potential to support precision medicine and improve long-term patient outcomes. AI, Artificial intelligence; COPD, Chronic obstructive pulmonary disease; RL, Reinforcement learning.)
